# Identification of Potential Novel Prognosis-Related Genes Through Transcriptome Sequencing, Bioinformatics Analysis, and Clinical Validation in Acute Myeloid Leukemia

**DOI:** 10.3389/fgene.2021.723001

**Published:** 2021-10-29

**Authors:** Jie Wang, Md. Nazim Uddin, Jian-ping Hao, Rong Chen, Yun-xia Xiang, Dai-qin Xiong, Yun Wu

**Affiliations:** ^1^ Department of Pharmacy, First Affiliated Hospital of Xinjiang Medical University, Urumqi, China; ^2^ School of Basic Medicine and Clinical Pharmacy, China Pharmaceutical University, Nanjing, China; ^3^ Institute of Food Science and Technology, Bangladesh Council of Scientific and Industrial Research (BCSIR), Dhaka, Bangladesh; ^4^ Department of Hematology, First Affiliated Hospital of Xinjiang Medical University, Urumqi, China; ^5^ Department of General Medicine, First Affiliated Hospital of Xinjiang Medical University, Urumqi, China

**Keywords:** Acute Myeloid Leukemia, transcriptome sequencing (RNA-seq), Competing endogenous RNA, prognosis-related genes, bioinformatics

## Abstract

**Background:** Acute Myeloid Leukemia (AML) is a complex and heterogeneous hematologic malignancy. However, the function of prognosis-related signature genes in AML remains unclear.

**Methods:** In the current study, transcriptome sequencing was performed on 15 clinical samples, differentially expressed RNAs were identified using R software. The potential interactions network was constructed by using the common genes between target genes of differentially expressed miRNAs with transcriptome sequencing results. Functional and pathway enrichment analysis was performed to identify candidate gene-mediated aberrant signaling pathways. Hub genes were identified by the cytohubba plugin in Cytoscape software, which then expanded the potential interactions regulatory module for hub genes. TCGA-LAML clinical data were used for the prognostic analysis of the hub genes in the regulatory network, and GVSA analysis was used to identify the immune signature of prognosis-related hub genes. qRT-PCR was used to verify the expression of hub genes in independent clinical samples.

**Results:** We obtained 1,610 differentially expressed lncRNAs, 233 differentially expressed miRNAs, and 2,217 differentially expressed mRNAs from transcriptome sequencing. The potential interactions network is constructed by 12 lncRNAs, 25 miRNAs, and 692 mRNAs. Subsequently, a sub-network including 15 miRNAs as well as 12 lncRNAs was created based on the expanded regulatory modules of 25 key genes. The prognostic analysis results show that *CCL5* and lncRNA *UCA1* was a significant impact on the prognosis of AML. Besides, we found three potential interactions networks such as lncRNA *UCA1*/hsa-miR-16-5p/*COL4A5*, lncRNA *UCA1*/hsa-miR-16-5p/*SPARC*, and lncRNA *SNORA27*/hsa-miR-17-5p/*CCL5* may play an important role in AML. Furthermore, the evaluation of the immune infiltration shows that *CCL5* is positively correlated with various immune signatures, and lncRNA *UCA1* is negatively correlated with the immune signatures. Finally, the result of qRT-PCR showed that *CCL5* is down-regulated and lncRNA *UCA1* is up-regulated in AML samples separately.

**Conclusions:** In conclusion, we propose that *CCL5* and lncRNA *UCA1* could be recognized biomarkers for predicting survival prognosis based on constructing competing endogenous RNAs in AML, which will provide us novel insight into developing novel prognostic, diagnostic, and therapeutic for AML.

## Introduction

Acute Myeloid Leukemia (AML) is one of the most common hematologic malignancies in adults and is characterized by a clonal expansion of myeloid abnormally differentiated blast cells (Short et al., 2018). According to the American Cancer Society’s estimates for leukemia in the United States for 2020, about 19,940 new cases are diagnosed with AML and more than 11,000 deaths from the disease (Bethesda, 2021). Although the overall survival rate for leukemia has steadily improved over time, recent study data suggest that older patient’s 5-year overall survival rate remains unsatisfactory (Hulegårdh et al., 2015; Silva et al., 2017). It should be noted that the data from the National Cancer Institute’s Surveillance, Epidemiology, and End Results (SEER) program report that the overall incidence trend for AML appears to be slowly increasing (Henley et al., 2020) and that this may be inextricably linked to differences in genetic and biological characteristics and an aging population (Rodriguez-Abreu et al., 2007; Lancet, 2018). Furthermore, despite significant advances in the genetic landscape of AML, standard treatments have not improved significantly over the past 30 years (Tyner et al., 2018). Therefore, it is necessary to explore the pathogenesis further and identify new biomarkers for AML, thus providing new perspectives for new therapies for AML.

AML is a hematological malignancy with complex pathogenesis because of gene mutations, karyotype abnormalities, and epigenetic changes that have been regarded as genetic risk factors that respect clinical outcomes (Medinger and Passweg, 2017). Despite discovering a large number of transcripts from previously overlooked non-protein-coding genomes, our understanding of non-coding RNA (ncRNA) that participates in this interaction is still limited. In most cases, many regulatory RNAs, including microRNAs (miRNAs) and long non-coding RNAs (lncRNAs), play key roles in developing the disease. MicroRNAs are generally delineated as a kind of single-strand, a small non-protein-coding molecule of 19–25 nucleotides, which display essential regulatory roles post-transcriptional regulation of messenger RNA (mRNA) targets (Dragomir et al., 2018). Thus, the most recent advances in genetics have found that microRNAs (miRNAs) play an essential role in the occurrence, development, and prognosis of AML. Various miRNAs have been identified as oncogenes or tumor suppressor genes (Starczynowski et al., 2011; Schwarzer et al., 2017; Wallace and O'Connell, 2017). Long non-coding RNAs (lncRNAs) are a group of non-coding RNAs of 200 bp or more that play critical roles in nuclear structure, regulation of gene expression, and various biological functions such as Immune Surveillance, Cancer Development, and Tumorigenesis maintenance (Rafiee et al., 2018; Izadirad et al., 2021; Liu et al., 2021). LncRNAs can also be regulated by sequestering and binding miRNAs and mRNAs. The competitive endogenous RNA (ceRNA) mechanism has attracted increasing attention since it was first proposed by Salmena et al. (Salmena et al., 2011). CeRNA is a complex post-transcriptional regulatory network using iRNA response elements (MREs) to compete for the binding of miRNAs, thereby implementing mutual control between mRNAs, lncRNAs, and miRNA (Wang C.-J. et al., 2019). Besides, many studies have recently reported that the CeRNA mechanism could play critical roles in the occurrence of a severe tumor, and some of the research focus on pathogenies of AML have noted that highly-upregulated LncRNA CCAT1, lncRNA KCNQ1OT1, and c-Myc act as ceRNA in the procession of AML (Chen et al., 2016; Cheng et al., 2020).

Herein, to reveal the potential interactions mechanism in the pathogenesis of AML and further development of novel recurrence prevention or treatment methods, we performed whole transcriptome sequencing and bioinformatic analysis on 15 clinical samples to screen the differentially expressed lncRNAs, miRNAs, and mRNAs in AML. Then we constructed the potential interactions network to reveal the possible mechanisms of the coregulation in lncRNA-miRNA-mRNA. Finally, we validated the expression of hub genes in independent clinical samples. Subsequently, our studies have enriched the biological functions of ceRNAs and further revealed critical regulatory networks in the pathogenesis of AML.

## Materials and Methods

### Patients and Samples Collection

Bone marrow specimens from ten patients who were newly diagnosed with AML at the Department of Hematology, The First Affiliated Hospital of Xinjiang Medical University, between January 2019 and September 2019. The Research Ethics Committee approved this study of The First Affiliated Hospital of Xinjiang Medical University (reference number: 220200320), and the written informed consent was obtained from all parents or guardians. Diagnosis of AML was according to the World Health Organization MICM (Morphology, Immunology, Cytogenetics, and Molecular biology) classification criteria. Detailed clinical records of each patient were collected, and the morphological diagnosis of each patient was re-confirmed by two independent morphologists.

The bone marrow samples of AML patients were collected before treatment. The Bone Marrow Mononuclear Cells (BMNCs) were isolated using lymphocyte separation liquid (Ficoll-Isopaque, Pharmacia) within 8 h in the library. The proportion of leukemic cells was determined using May-Grünwald-Giemsa stained cell centrifuge preparations and light microscopy, and the BMNCs were harvested contained at least 90% blasts in each separation. Total cellular RNA was isolated using TRIzol (Invitrogen, Carlsbad, CA, United States ) then stored at −80°C. The clinical data were retrieved with the last follow-up on September 30th, 2019, and peripheral blood mononuclear cells from five anonymized healthy volunteers were included as control samples.

### Whole Transcriptome Sequencing

The qualified total RNA samples from 10 AML patients and 5 healthy volunteers were used for whole transcriptome sequencing. Novogene Bioinformatics Technology Co., Ltd performed transcriptome analysis. A total amount of 3 μg total RNA per sample was used as input material for the RNA sample preparation. Using the NEBNext® UltraTM RNA Library Prep Kit for Illumina® (NEB, United States ) and the NEBNext® Multiplex Small RNA Library Pre-Set for Illumina® (NEB, United States ) and generate sequencing libraries following the manufacturer’s recommendations, and then add the index code to the attribute sequence of each sample. According to the manufacturer’s instructions, clustering index-coded samples was carried out on the cBot cluster generation system using TruSeq PE Cluster Suite v3-cBot-HS (Illumia). After DNA clone cluster generation, the prepared libraries were sequenced on Illumina Hiseq 2,500/2,000 platform, miRNA sequencing generated 50 bp single-end reads (reads), and transcriptional profiling generated 125 bp/150 bp double-end reads. Principal Component Analysis (PCA) of transcripts was performed to describe the relationships among different samples.

### Data Analysis

After data quality control, reads mapping to the reference sequence, and quantification of gene expression level, the transcriptome difference analysis was performed on 10 AML patients and 5 healthy controls. The “DESeq” package(Anders and Huber, 2010) in the R v4.0 software screen for differentially expressed genes in AML samples and healthy controls. The lncRNA or mRNA with |log2 FC|>2.0 and corrected *p* <0.01 were considered as differentially expressed lncRNA (DElncRNA) or differentially expressed mRNA (DEmRNA) between AML samples and healthy controls; The differentially expressed miRNA (DEmiRNA) were also screened out with |log2 FC|>2 and corrected *p* <0.01. FDR (false discovery rate, FDR) (Benjamini-Hochberg method) is used for multiple test corrections due to many genes analyzed. When FDR<0.05, it is considered to be statistically different. We generated a heatmap and volcano map using the ggplot2 packages in the R platform for the obtained differentially expressed RNAs.

### Construction of Potential Interactions Network

We constructed a potential interactions network based on the theory that lncRNAs can affect miRNAs and act as miRNA sponges to regulate mRNAs further. Predicting the target genes of DEmiRNA was performed by the RNAInter database (www.rna-society.org/raid/) (Lin et al., 2020), then retained the common genes from predicted target genes intersected with DEmRNA or DElncRNA separately. Finally, the common genes with regulatory relationship scores> 0.5 were screened to construct the ceRNA regulatory network. Cytoscape (version 3.8.0) (Shannon et al., 2003) was utilized to build the lncRNA-miRNA-mRNA ceRNA network.

### GO and KEGG Enrichment Analysis

Gene Ontology (GO) analysis of differentially expressed genes was implemented using the “clusterProfiler” package (Yu et al., 2012) in the R program. GO terms with corrected *P*-values less than 0.05 were considered significantly enriched for differentially expressed genes.

KEGG is a database resource for obtaining the high-level function and usefulness of biological systems, including cells, organisms, and ecosystems, from molecular-level information, particularly the large-scale molecular datasets produced by high-throughput experimental technologies like genome sequencing (http://www.genome.jp/kegg/) (Kanehisa et al., 2021). We also used the “clusterProfiler” package of the R program to test the statistical enrichment of differential expression genes in KEGG pathways.

### Identification of Hub Genes in the Potential Interactions Network

To identify the hub genes in the potential interactions network, we first constructed the Protein-Protein Interaction (PPI) network in the STRING database (Szklarczyk et al., 2017). Twenty-five hub genes were then screened with the Maximal Clique Centrality (MCC) using the cytoHubba plugin (Chin et al., 2014) in Cytoscape (version 3.8.0). Finally, using the selected 25 hub genes to extend the whole ceRNA network and the potential core interactions in the regulatory network were determined.

### Verified the Prognostic Signature of Hub Genes in the Potential Interactions Network

To further study the prognosis of lncRNAs in AML, using the downloaded survival data(n = 151) and phenotype data (n = 697) of TCGA-LAML from the UCSC Xenadatabase (http://xena.ucsc.edu/) (Goldman et al., 2020). Besides, we validated the impact of specific hub genes and clinical characters on the overall survival time of AML from the TCGA database (n = 151) (Weinstein et al., 2013). Moreover, we have analyzed the risk factor in the univariate *Cox* risk proportional model, multivariate *Cox* risk proportional model, and the Nomogram, respectively (Liu et al., 2020; Lee et al., 2021). “Survival,” “Survminer,” and “rms” programs in R software (version 4.0.3) were used to analyze and visualize the results. Survival analysis uses the *Log-rank* test for hypothesis testing. A *Cox* proportional regression model was used to estimate the essential gene’s hazard ratio and 95% confidence interval. *p* <0.05 is considered statistically significant. Nomogram was constructed to provide the certain influence of genes and clinical factors on the prognosis.

### ROC Curve Analyzing the Diagnostic Value of the Hub Genes in an Independent Cohort Study

To determine the diagnostic value of specific mRNA and lncRNA in AML patients, we used the Receiver Operating Characteristic Curve (ROC) to analyze the relationship between gene expression and disease diagnosis. Using gene expression from an independent microarray data set (GSE114868) (Yang et al., 2017), we calculated the areas under the ROC curve (AUC) of each gene to quantify the prediction accuracy. The Wilcoxon signed-rank test was applied to calculate the *p*-value of each gene, and we compared the expression profiles of the hub genes between AML and healthy. Additionally, we defined the area under the curve (AUC) according to the general rules of statistical analysis for the assessment of the diagnostic value of genes, including a cut-off value of 0.5 to assess the diagnostic value of genes. Commonly, the AUC of genes in ROC curves between 0.5 and 0.7 considered with general diagnostic accuracy for identifying AML, the AUC of genes reached 0.7–0.9 considered with better diagnostic accuracy, and the closer the AUC is to 1, the genes will define with idealistic diagnosis value.

### Evaluation of the ESTIMATE Scores and Immune Signature Enrichment Levels

ESTIMATE is an algorithmic tool based on the R package for predicting tumor purity, which uses the gene expression profiles of 141 immune genes and 141 stromal genes to generate ESTIMATE scores, immune scores, and stromal scores (Yoshihara et al., 2013). The presence of infiltrated immune cells and stromal cells in tumor tissues was calculated using related gene expression matrix data, represented by immune and stromal scores.

We also identified the immune signature’s enrichment level in the TCGA-LAML sample as the Single-Sample Gene-Set Enrichment Analysis (ssGSEA) score (Hänzelmann et al., 2013). The gene set contains the collection of all marker genes of an immune signature. We included 11 immune signatures: B cell, CD8^+^ T cells, CD4^+^ regulatory T cells, NK cells, Tregs, MDSC, TAM, macrophages, M2 macrophages, CAFs, and Th17. The threshold is the absolute value of *R* is not less than 0.30 and *P* <0.05.

### qRT-PCR Validated the Expression of Critical Genes in Initial Diagnosis Patients With AML.

To verify the expression of key genes in clinical samples, we further validated the expression levels of key genes in bone marrow mononuclear cells from 12 newly diagnosed AML patients (confirmed by WHO-AML criteria (Arber et al., 2016), excluding AML-M3 cases, not receiving any treatment) using qPCR. The clinical samples were collected from bone marrow using heparinized tubes, and 1.077 g/ml Ficoll-Isopaque (Pharmacia) density gradient centrifugation was used to isolate bone marrow mononuclear cells, and cell proportions were estimated by light microscopy. Isolated samples contained 2 to 10 million cells were stored in TRIzol (Invitrogen, Carlsbad, CA, United States) and immediately frozen at –80 C. Peripheral blood mononuclear cells from 12 anonymous healthy volunteers were used as control samples.

CDNA was quantified using a reverse transcription kit (K1622, Thermo Fisher Scientific, United States ), and the expression levels of *CCL5* and lncRNA *UCA1* using SYBR Green Master Mix (SYBR GREEN, Beijing, China). The housekeeping gene GAPDH was used as an internal control. The primers were synthesized by Shanghai Biotechnology Co., Ltd (Table 1). qRT–PCR was performed on ViiATM 7 system software (Thermo Fisher Scientific, ABI7500, United States). The results were normalized to the expression of *GAPDH* and expressed as fold change (2^−ΔΔCT^). The qRT-PCR experiment on each clinical sample with three biological replicates.

**TABLE 1:** The primers for qRT-PCR.

Target	Sequence (5' - 3′)
*UCA1* (human) -RT-F	GCC​GAG​AGC​CGA​TCA​GAC​AAA​C
*UCA1* (human) -RT-R	AAC​GGA​TGA​AGC​CTG​CTT​GGA​AG
*CCL5* (human) -RT-F	CAG​CAG​TCG​TCC​ACA​GGT​CAA​G
*CCL5* (human) -RT-R	TTT​CTT​CTC​TGG​GTT​GGC​ACA​CAC
*GAPDH* (human) -RT-F	AGA​AGG​CTG​GGG​CTC​ATT​TG
*GAPDH* (human) -RT-R	AGG​GGC​CAT​CCA​CAG​TCT​TC

### Statistical Analysis

Statistical analysis and visualization were achieved using R Studio (R version 4.0.2) and GraphPad Prism 8.3 software (GraphPad Software, Inc., La Jolla, CA, United States ). The mRNA and lncRNA expression data set and the overall survival information was profiled using the univariate and multivariate *Cox* proportional regression model. The prognosis results were presented as Kaplan-Meier survival curves. Bilateral unpaired t-tests were used to compare the differences in the expression level of hub genes. *P* < 0.05 was considered as a statistically significant difference.

## Results

### Identification of Differentially Expressed RNAs

**FIGURE 1:**
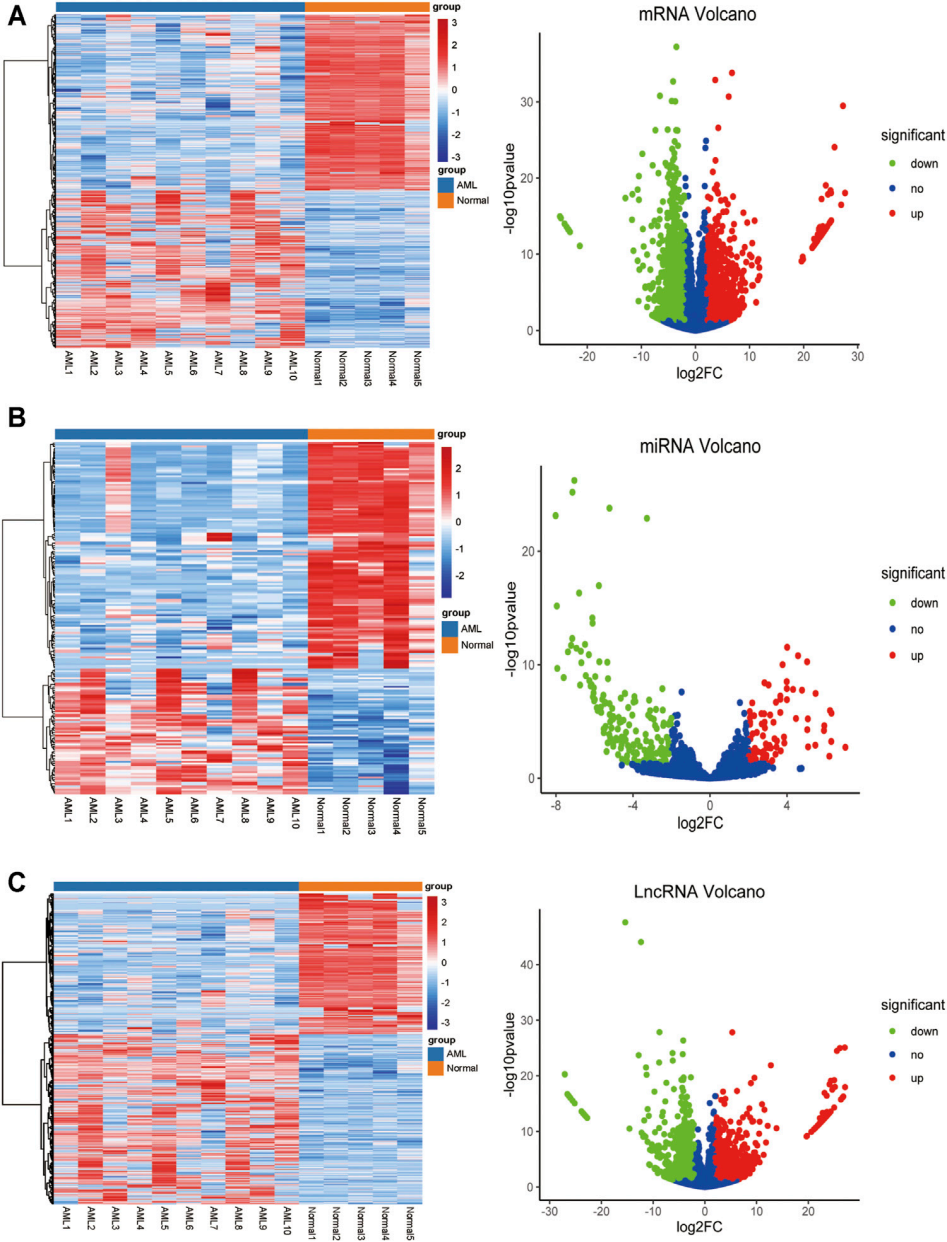
The heatmap and volcano plot of DEmRNAs, DEmiRNAs, and DElncRNAs **(A)** The heatmap and volcano plot of DEmRNAs **(B)** the heatmap and volcano plot of DEmiRNAs **(C)** the heatmap and volcano plot of DElncRNAs. The heatmap shows the clustering results of mRNA, miRNA, and lncRNA expression between AML and normal samples. The volcano plot shows the differential analysis results of transcriptome sequencing. The green and red dots indicate the down-regulated and up-regulated differentially expressed genes that reached |log2 FC|> 2 and *P*-value <0.05, respectively, and the blue dots indicate that the differentially expressed genes are not meet the screening criteria.

To identify differentially expressed mRNAs, lncRNAs, and miRNAs between 10 AML and five control patients differentially, total BMNCs were collected for whole transcriptome sequencing. The clinical information of the samples is shown in Supplementary Table S1. The workflow of the analysis procedure in this study is shown in Supplementary Figure S1. The expressed transcripts revealed substantial differences between different groups (Supplementary Figure S2). Whole transcriptome sequencing analysis detected a total of 63,036 genes, the differentially expressed genes between AML and normal samples were identified after background correction and normalization. Furthermore, 1584 DElncRNAs (863 upregulated and 721 downregulated), 233 DEmiRNAs (85 upregulated and 148 downregulated), and 2,217 DEmRNAs (1046 upregulated and 1171 downregulated) were screened out with |log2 FC| > 2 and *p*-value < 0.05 (Supplementary Tables S2–4). All the differentially expressed lncRNAs, miRNAs, and mRNAs were presented using hierarchical clustering heat maps and volcano plots in Figures 1A–C.

### Prediction of miRNA Targets and Construct the Potential Interactions Network

**FIGURE 2:**
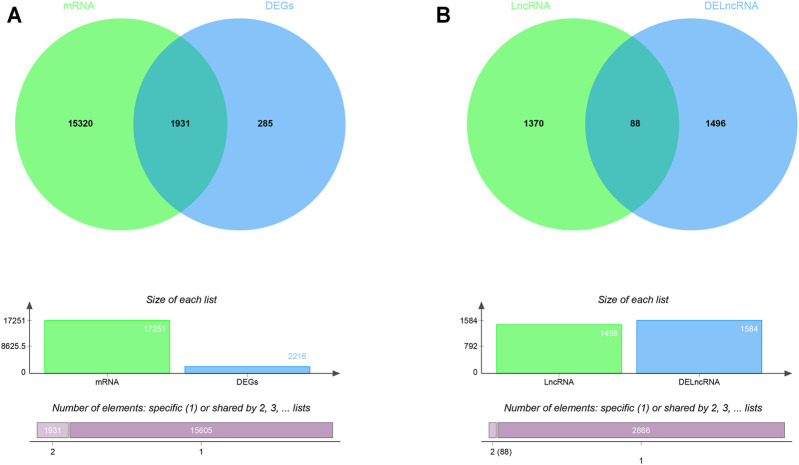
The Venn diagram of interacted mRNAs and lncRNAs **(A)** The Venn diagram of interacted mRNA between DEmRNA and DEmiRNA predicted targets **(B)** The Venn diagram of interacted lncRNA between DElncRNA and DEmiRNA predicted targets.

The RAID v2.0 database was used to predict the DEmiRNA-targeted mRNAs and lncRNAs, of which there are 35 experimentally validated and computationally predicted RNA interactome resources (such as starBase v2.0, miRTarBase, miRDB, et al.) were integrated into the RAID v2.0 database. It provides us with an advanced tool for a comprehensive understanding of the regulatory procession’s biological functions and molecular mechanisms of cellular RNAs. Thus, 17,251 mRNAs and 1458 lncRNAs were predicted by 233 DEmiRNA separately. Then, we applied the predicted target genes overlapped with differentially expressed LncRNA and mRNA, respectively. As a result, 71 DEmiRNAs, 1931 interactions between DEmRNAs and predicted target mRNAs, and 88 interactions between DElncRNAs and predicted target lncRNAs were identified (Figures 2A,B). Finally, using Cytoscape software (version 3.8.0), 14 lncRNAs, 25 miRNAs, and 692 mRNAs with a score of >0.5 were used to construct the potential interactions network (Supplementary Figure S3).

### Functional Enrichment Analyses

**FIGURE 3:**
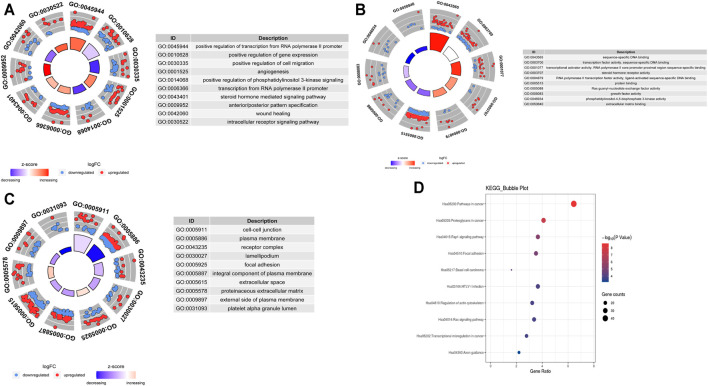
GO and KEGG analysis of hub mRNAs in the potential interactions network **(A)**Top 10 biological processes **(B)** Top 10 cellular components **(C)** Top 10 molecular functions; The circles in the outer circle of the circle plot show the logFC values of genes for each term using scatter. Red circles indicate upregulation, and blue indicates downregulation **(D)** Results of the top 10 enriched KEGG pathways.

To explore the biological effects of 692 mRNAs in the potential interactions, we performed GO analysis and KEGG pathway analysis using the “clusterProfiler” package in the R program. The biological process of mRNAs in the potential interactions is mainly involved in positive regulation of both transcription from RNA polymerase II promoter, gene expression, and cell migration, also take part in the angiogenesis, phosphatidylinositol 3-kinase signaling pathway, and steroid hormone-mediated signaling pathway (Figure 3A). The cellular component of mRNAs in the potential interactions network is mainly involved in cell-cell junction, plasma membrane, and receptor complex (Figure 3B). The mRNA’s molecular function in the potential interactions involved primarily sequence-specific DNA binding, transcription factor activity, and sequence-specific DNA binding (Figure 3C). The top 10 GO function analysis results are shown in Supplementary Table S5. KEGG results show that the mRNAs in the potential interactions mainly enriched in various signaling pathways in cancer (Figure 3D), and the top 20 KEGG enrichment results were shown in Supplementary Table S6.

### Identification of Hub Genes in the Potential Interactions Network

**FIGURE 4:**
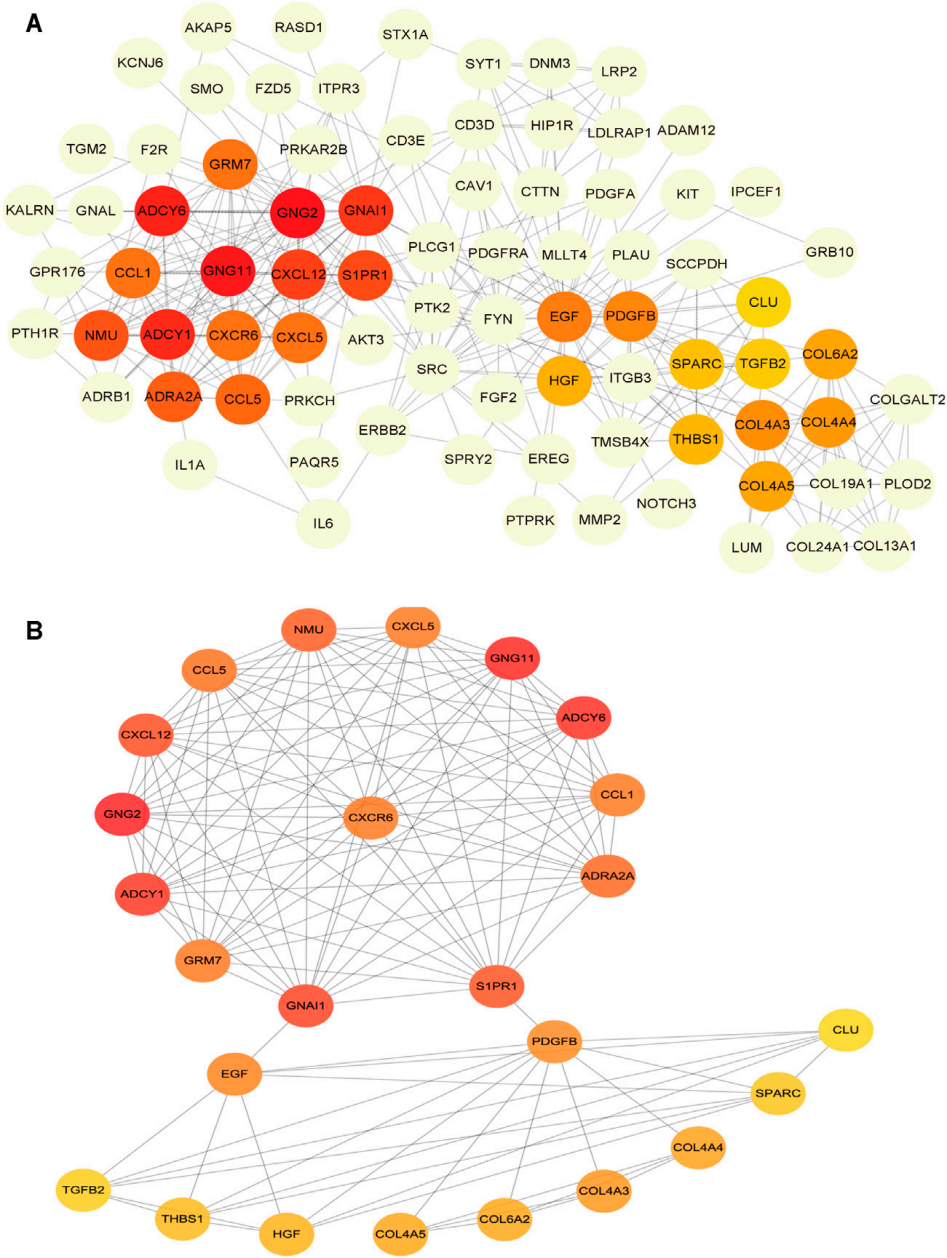
Construction of protein-protein interaction (PPI) network in the potential interactions network **(A)** The hub genes PPI network **(B)** The top 25 hub genes interactions. The darker color (red) of the mRNAs represents the gene with a higher centrality in the interaction network.

**FIGURE 5:**
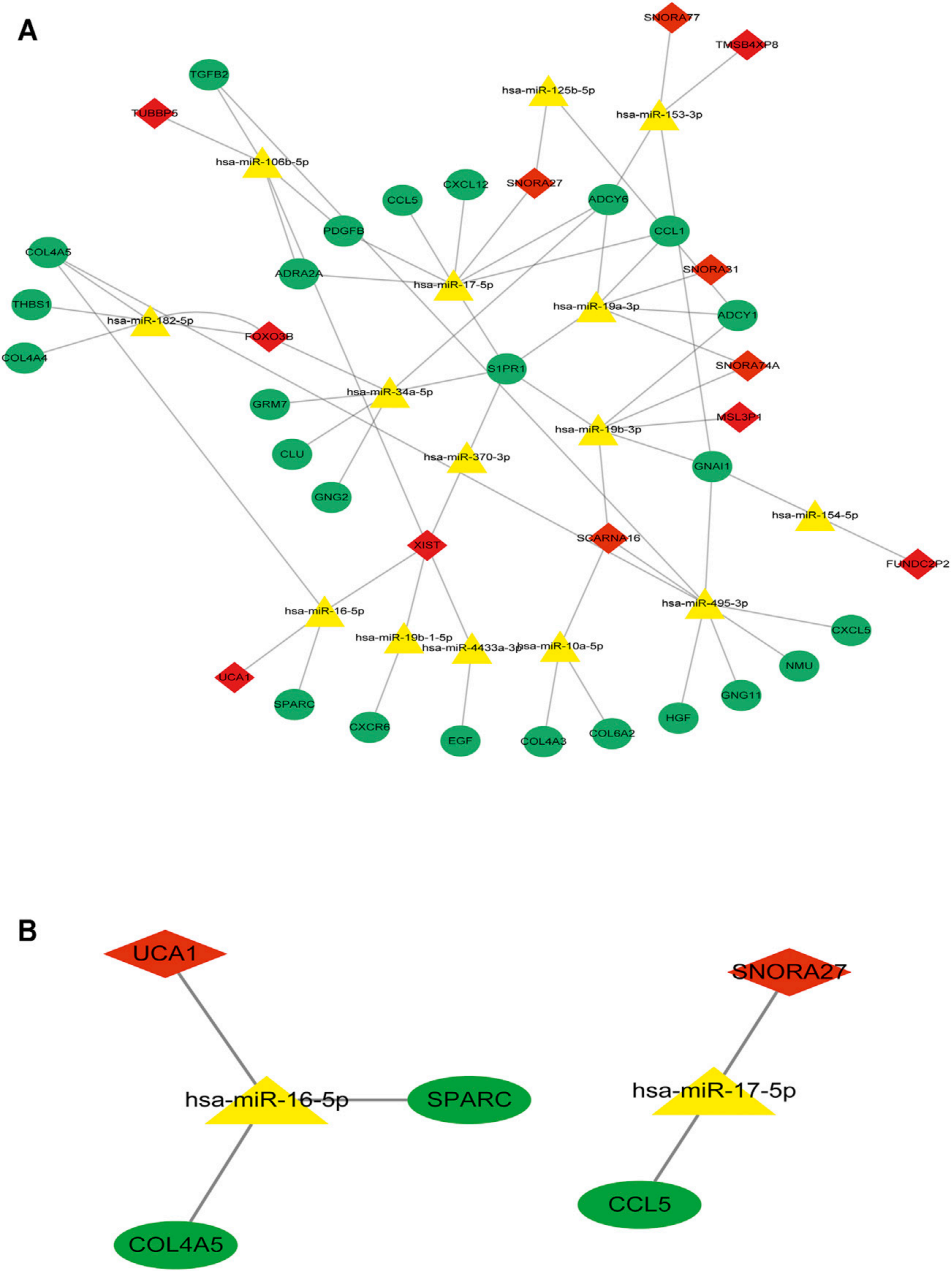
The hub genes interacted with miRNAs and lncRNAs in the potential interactions network **(A)** The potential interactions network of lncRNA-miRNA-mRNA regulation relationships. The green dot represents hub mRNAs in the potential interactions network; the yellow triangle represents the interacted miRNAs in the potential interactions network; the red rhombus represents the interacted lncRNAs in the potential interactions network **(B)** The prognosis-related genes sub-network of lncRNA-miRNA-mRNA regulatory relationships.

To determine the hub mRNAs in the potential interactions network, we used the STRING database to construct the Protein-Protein Interaction (PPI) network (Figure 4A). Besides, using the cytoHubba plugin in Cytoscape software, we screened and visualized 25 hub genes in the PPI network (Figure 4B). Finally, the 25 mRNAs interacted with 15 miRNAs, and 12 lncRNAs were extended in the potential interactions network (Table 2, Figure 5A). The prognosis-related genes sub-network further showing lncRNA-miRNA-mRNA regulatory relationships (Figure 5B).

**TABLE 2:** The list of hub mRNAs, interacted miRNAs, and lncRNAs in the potential interactions network.

Type of genes	Genes names
mRNAs	*GNG2, GNG11, ADCY6, ADCY1, GNAI1, CXCL12, S1PR1, NMU, ADRA2A, CCL5, CCL1, CXCL5, CXCR6, GRM7, EGF, PDGFB, COL4A3, COL4A4, COL6A2, COL4A5, HGF, THBS1, SPARC, TGFB2, CLU*
miRNAs	*hsa-miR-34a-5p, hsa-miR-495-3p, hsa-miR-19a-3p, hsa-miR-17-5p, hsa-miR-153-3p, hsa-miR-19b-3p, hsa-miR-125b-5p, hsa-miR-154-5p, hsa-miR-370-3p, hsa-miR-106b-5p, hsa-miR-19b-1-5p, hsa-miR-4433a-3p, hsa-miR-10a-5p, hsa-miR-182-5p, hsa-miR-16-5p*
lncRNAs	*FOXO3B, SCARNA16, SNORA31, SNORA74A, SNORA27, TMSB4XP8, SNORA77, MSL3P1, FUNDC2P2, XIST, TUBBP5, UCA1*

### Prognostic Evaluation of Hub Genes in the Potentially Interacted Network

Based on the TCGA-LAML clinical information from the UCSC database (n = 151), we conducted prognostic verification on selected essential mRNAs and lncRNAs to verify the screened hub genes’ prognostic value.

**FIGURE 6:**
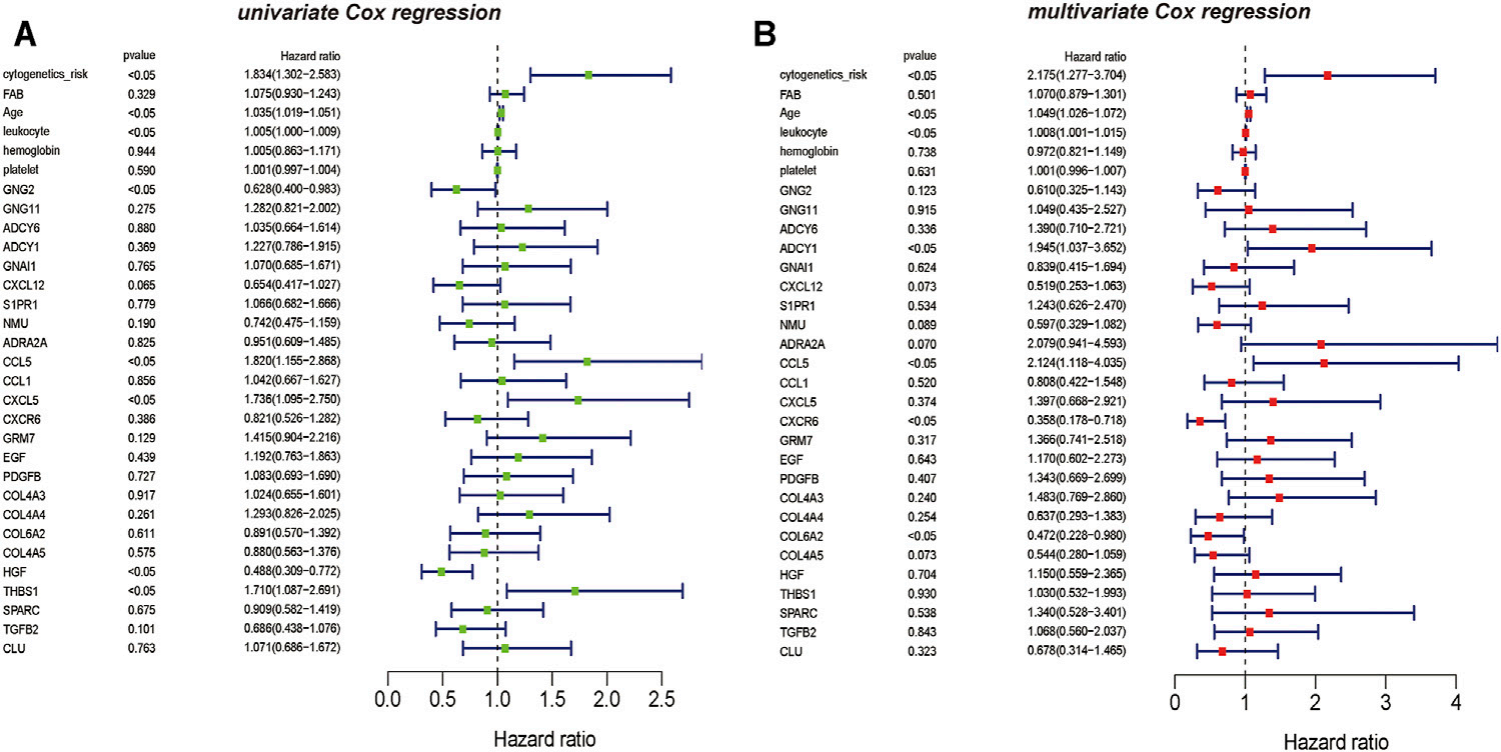
The univariate and multivariate *Cox* regression analysis on hub mRNAs **(A)** The result of mRNAs *Cox* regression analysis showed that the clinical information such as cytogenetics risk, age, and leukocyte could be identified as the risk factors on AML, the genetic features including *CCL5*, *CXCL5*, and *THBS1* also could be recognized as the risk factors on AML, and *GNG2* is the protective factor on AML **(B)** The result of multivariate *Cox* regression analysis also showed that the clinical information such as cytogenetics risk, age, and leukocyte could be identified as the risk factors on AML, and the genetic features including *ADCY1*, *CCL5* could be recognized as the risk factors on AML. However, *CXCR6* and *COL6A2* are the protective factors on AML.

The results of univariate *Cox* regression analysis showed that critical mRNAs such as *GNG2, CCL5, CXCL5,* and *HGF* showed as a protective factor in AML, but *THBS1* is selected as risk factors affecting the prognosis of AML patients (*p* <0.05) (Figure 6A). Besides, the results of multivariate Cox regression analysis showed that the critical mRNAs *ADCY1, CCL5* are selected as risk factors affecting the prognosis of AML patients, but *CXCR6* and *COL6A2* are showed as protective factors in AML (*p* <0.05) (Figure 6B).

**FIGURE 7:**
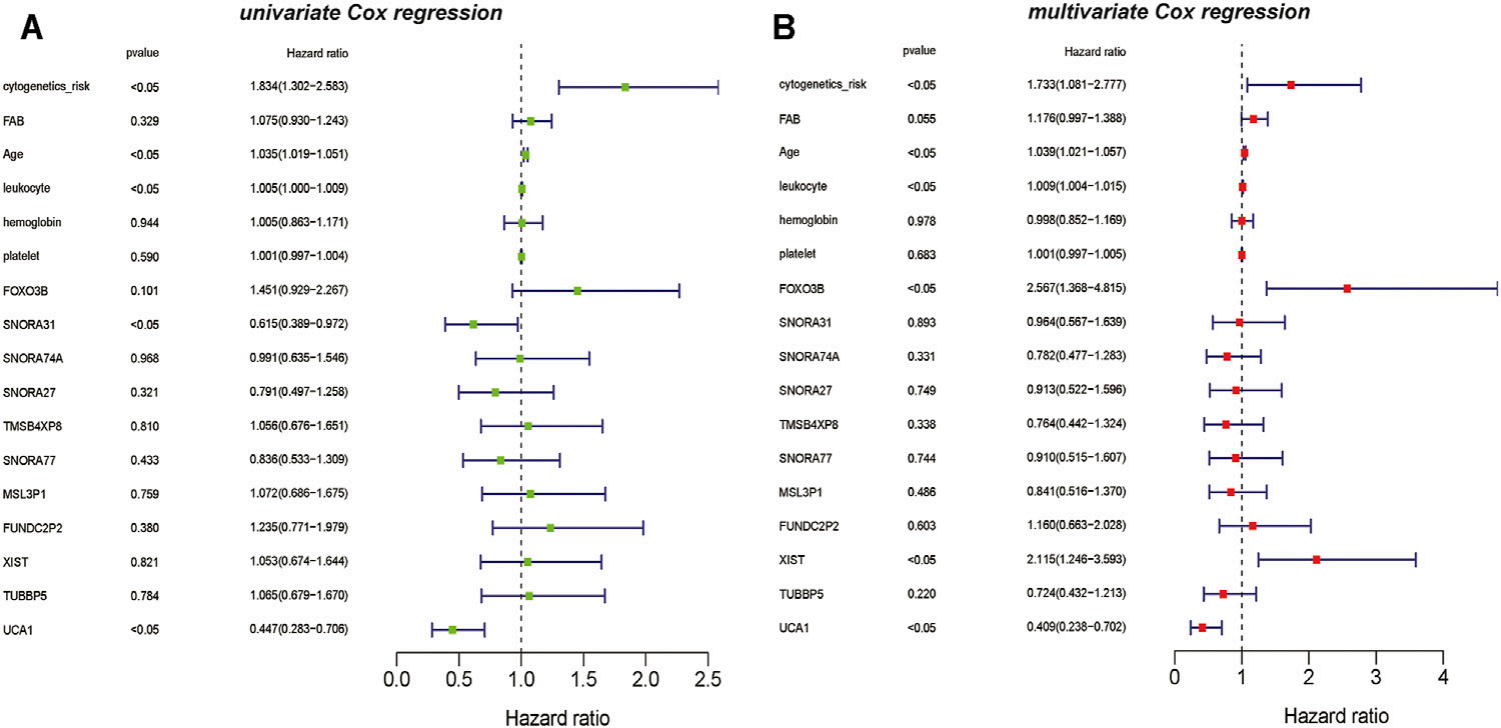
The univariate and multivariate *Cox* regression analysis on lncRNAs **(A)** The result of univariate *Cox* regression analysis showed that the clinical information such as cytogenetics risk, age, and leukocyte could be identified as the risk factors on AML, and the genetic features including lncRNA *SNORA31* and lncRNA *UCA1* are recognized as the protective factors in AML **(B)** The result of multivariate *Cox* regression analysis also showed that the clinical information such as cytogenetics risk, age, and leukocyte could be identified as the risk factors on AML, the genetic features including lncRNA *FOXO3B,* lncRNA *XIST* could be recognized as the risk factors on AML. However, lncRNA *UCA1* is the protective factor on AML.

Simultaneously, based on the results of univariate *Cox* regression analysis for vital lncRNAs, *UCA1* and *SNORA31* are showed as the protective factors in AML(*p* <0.05) (Figure 7A). However, the results of multivariate Cox regression analysis showed that the vital lncRNAs such as *FOXO3B* and *XIST* are the risk factors affecting the prognosis of AML patients, instead of lncRNA *UCA1* is still the protective factor in AML (*p* <0.05) (Figure 7B).

Interestingly, the results of the univariable and multivariable analysis showed the difference between univariate and multivariate analyses. Such as *HGF* and *THBS1*, the univariate analysis results for *HGF* and *THBS1* were significant, but they were not significant in the multivariate analysis. And similar results occurred in the analysis of lncRNAs, lncRNA *SNORA31,* with significant results in multivariable analysis but not in univariable analysis. Moreover, *FOXO3B* and *XIST* were known to be associated with oxidative stress and upregulation of *MYC* expression also showed a biased result. They were not shown significantly in univariate analysis but showed a significant prognostic effect in multivariate analysis. Thus, considering the complexity of AML disease, we only selected results that remained consistent in univariate and multivariate analyses for further study. And this finding also suggests that these important genes may act different biological functions in AML through synergy with other molecules.

**FIGURE 8:**
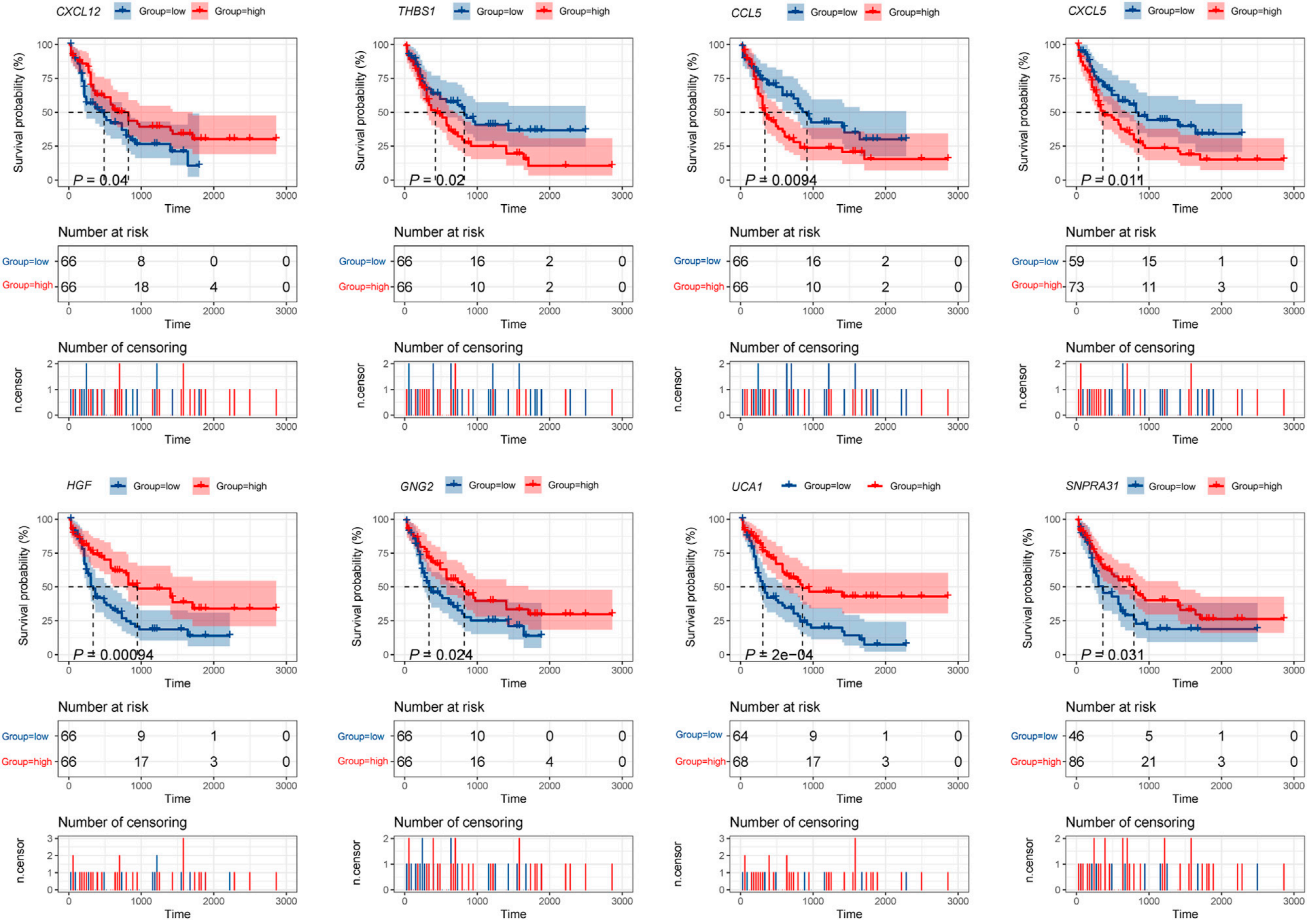
*K-M* survival analysis of hub mRNAs and lncRNAs in the potential interactions network. The result showed that high expression of *THBS1*, *CCL5,* and *CXCL5* were significantly associated with poor prognosis in AML, while high expression of *CXCL12*, *HGF*, *GNG2*, lncRNA *UCA1,* and lncRNA *SNPRA31* were significantly associated with good prognosis (*p* <0.05).

**FIGURE 9:**
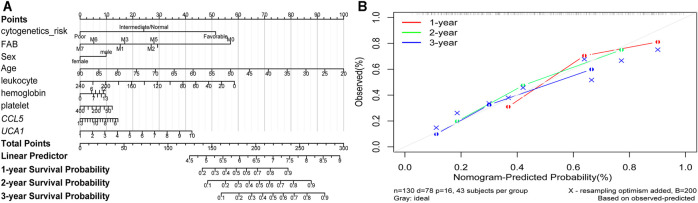
The Nomogram of risk factors influencing the prognostic outcome **(A)** The 1, 2, and 3 years overall survival of AML was predicting in Nomogram **(B)** The calibration curve for the overall survival nomogram. A grey diagonal line shows the ideal Nomogram, the red, green, and blue lines represent the nomograms observed at 1, 2, and 3 years, respectively.

Furthermore, the *K-M* survival analysis result showed that AML patients with high expression of *THBS1, CCL5,* and *CXCL5* could have a poor prognosis (*p* <0.05), but patients with a high expression level of *CXCL12, HGF, GNG2,* lncRNA *SNPRA31,* and lncRNA *UCA1* could have a good prognosis (*p* <0.05) (Figure 8). It is worth noting that, combining these prognostic analysis results, we confirmed that *CCL5* and lncRNA *UCA1* showed consistent results in either *Cox* regression or *K-M* analysis. Therefore, we have independently expanded the interactions of these two genes based on the previous finding and propose that the three potential interactions regulatory networks: lncRNA *UCA1*/hsa-miR-16–5p/*COL4A5*, lncRNA *UCA1*/hsa-miR-16–5p/*SPARC*, and lncRNA *SNORA27*/hsa-miR-17–5p/*CCL5* may play an essential role in AML. Finally, we used the Nomogram to evaluate the probability of these two hub genes influencing the prognostic outcome (Figures 9A,B).

### The Diagnostic Value of Identified Hub Genes

**FIGURE 10:**
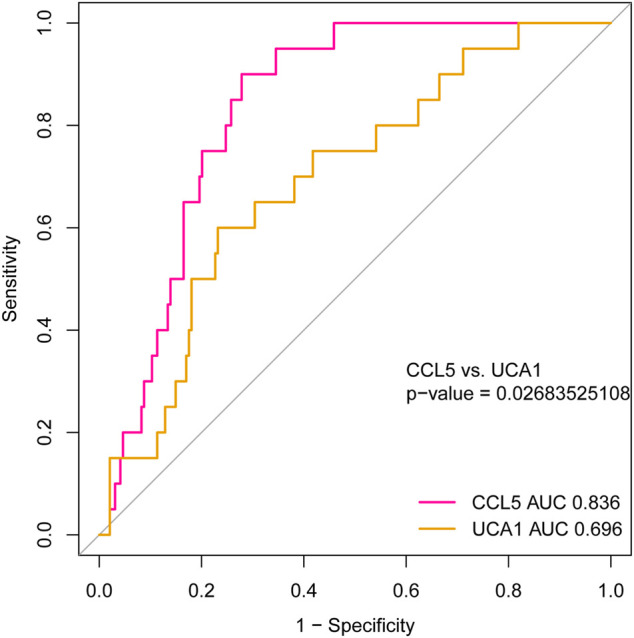
The ROC curve for analyzing the diagnostic value of hub mRNAs and lncRNAs. The result showed that both *CCL5 (*AUC = 0.836, 95% CI: 0.773–0.899) and lncRNA *UCA1* (AUC = 0.696, 95% CI: 0.581–0.811) have good diagnostic value in AML. Furthermore, the diagnostic value’s comparison results between *CCL5* and lncRNA *UCA1* represent better diagnostic sensitivity and specificity in *CCL5* (*P* <0.05).

To investigate the hub genes’ diagnostic value, which may be recognized as new and potential biomarkers for AML, we used the ROC curve analysis to assess the hub genes’ diagnostic ability. Based on the independent cohort study’s expression data analysis, we found that both *CCL5* and lncRNA *UCA1* have remarkable diagnostic capability. As represented in Figure 10, the AUC values for *CCL5* and lncRNA *UCA1* were 0.836 (95% Confidence Interval [CI], 0.773–0.899) and 0.696 (95% Confidence Interval [CI], 0.581–0.811) separately. Furthermore, we compared the diagnostic value of *CCL5* and lncRNA *UCA1* through the ROC curve and found *CCL5* had more diagnostic sensitivity and specificity for AML (*p* <0.05).

### Analyzing the Correlations of Hub Genes Expression Levels with Immune Score and Stroma Score in TCGA-LAML Cohort

**FIGURE 11:**
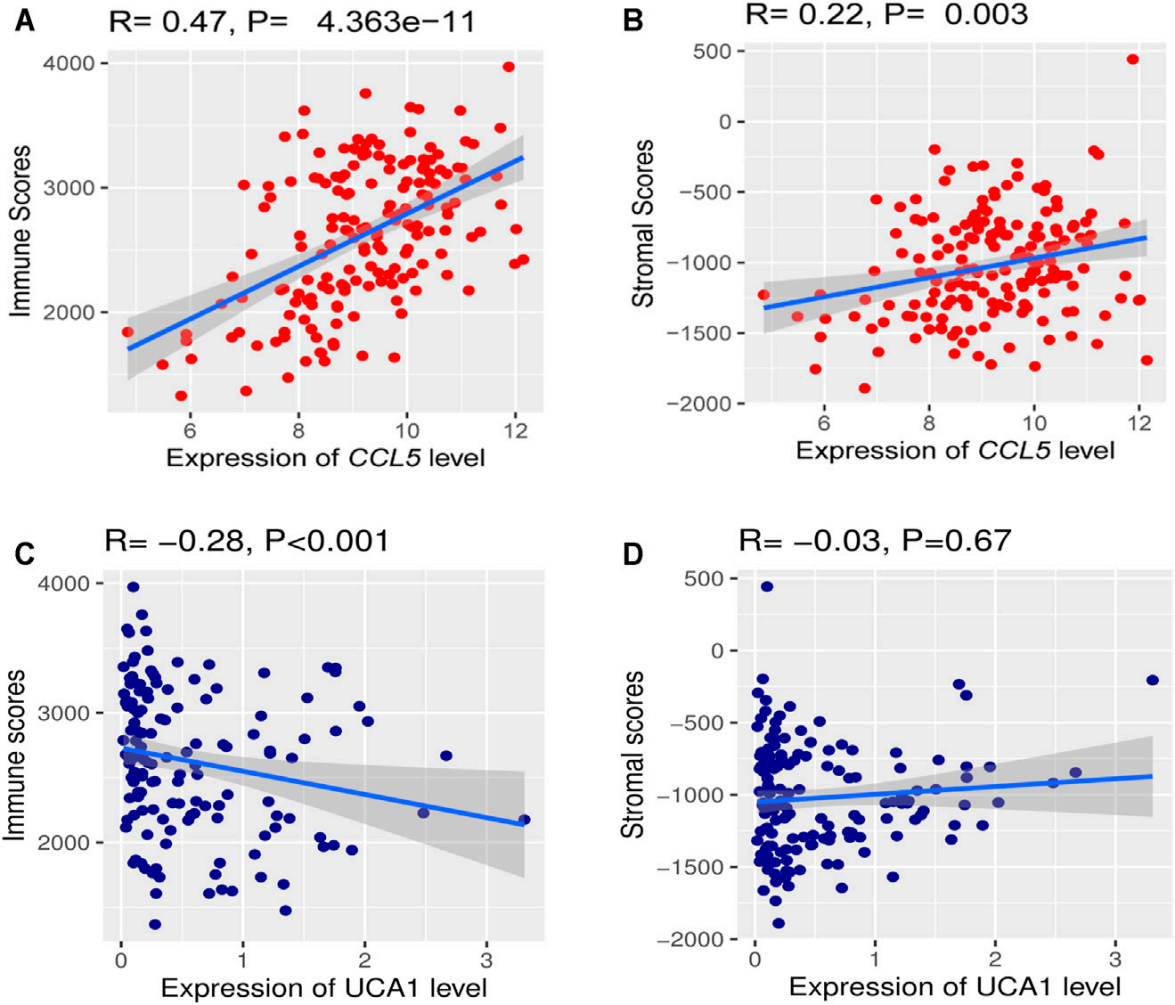
The correlations of hub gene expression levels with the tumor microenvironment (TME) **(A)** A positive correlation of *CCL5* expression (Normalized expression levels) and immune and stromal scores **(B)** Negative correlation between lncRNA *UCA1* expression (Normalized expression levels) and immune score.

We investigated the hub gene expression level’s association with immune and stroma scores in the TCGA-LAML cohort to analyze further the correlations of hub gene expression levels with immune and stroma scores. Interestingly, we found that the expression levels (log2 transformation) of *CCL5* were positively correlating with the immune score (Spearman’s correlation test, *R* = 0.47, *P* = 4.363e^−11^) and stroma score (Spearman’s correlation test, *R* = 0.22, *P* = 0.003) (Figure 11A). Besides, the expression levels (log2 transformation) of lncRNA *UCA1* were negatively correlating with the immune score (Spearman’s correlation test, *R* = -0.28, *p* < 0.001), but not significantly related to the stroma score (Figure 11B). This result prompted that the expression levels of *CCL5* and lncRNA *UCA1* are associated with the immune and stromal activity in AML.

### Association of Critical Genes Expression Level with Immune Signatures in AML

**FIGURE 12:**
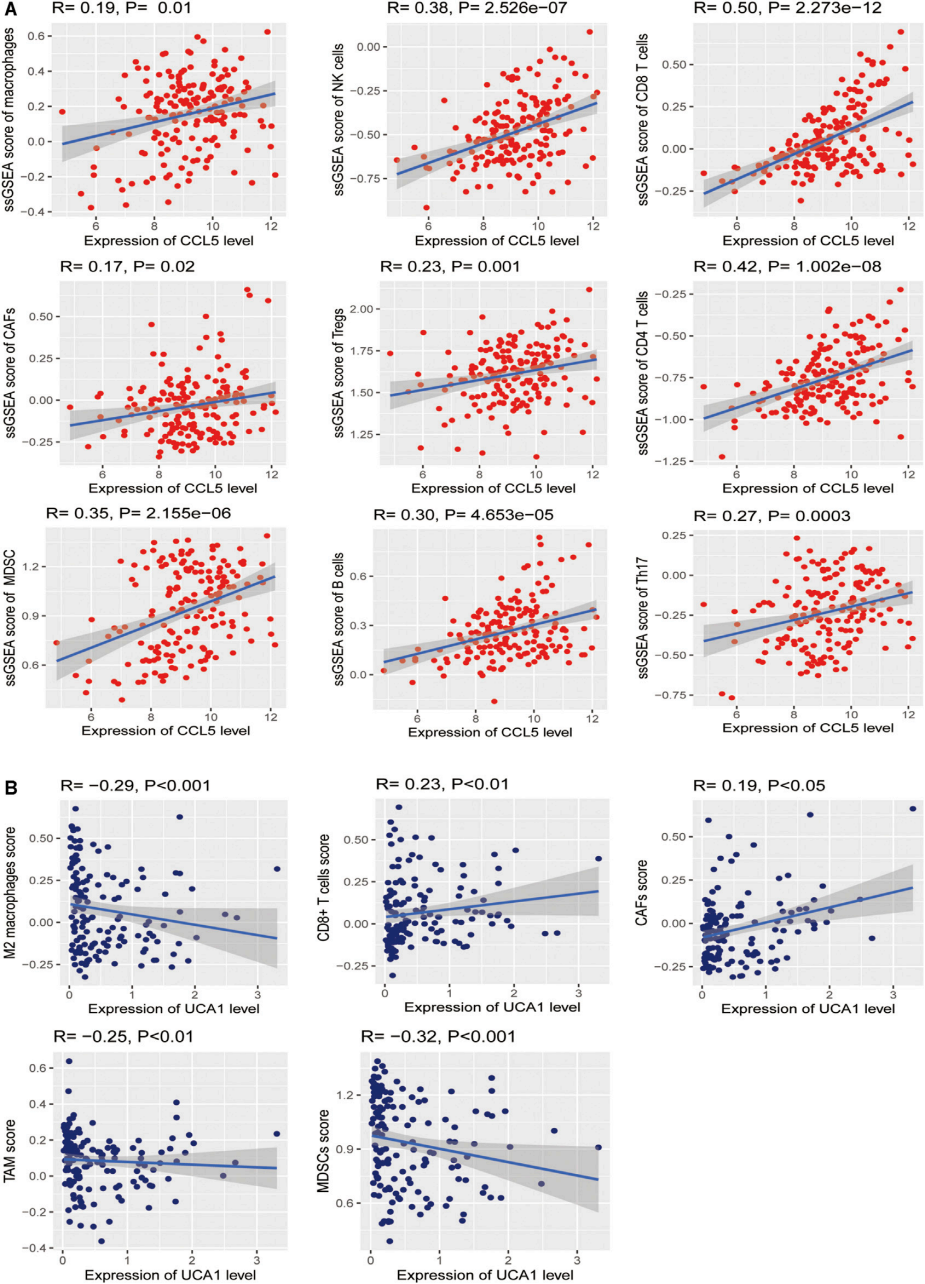
Association between hub genes and immune signatures in AML **(A)** The expression of *CCL5* (Normalized expression levels) exhibits a significant positive correlation with ten immune cells (Macrophages, NK cells, CD8^+^ T cells, CAFs, Treg, CD4^+^ regulatory T cells, MDSC, TAM, B cells, and Th17). The results of Spearman’s correlation test are shown as *P* <0.05 **(B)** The expression of *UCA1* (Normalized expression levels) exhibits a significant positive correlation with two immune cells (CAFs and CD8 cells); besides, *UCA1* also indicates a significant negative correlation with three immune cells (TAM, MDSC, and M2 macrophages). The results of Spearman’s correlation test are shown as *P* <0.05.

Based on the analysis in the TCGA-LAML cohort, we further found significant positive correlations of the *CCL5* expression levels with the enrichment levels (ssGSEA scores) of immune cells, including Macrophages, NK cells, CD8^+^ T cells, CAFs, Treg, CD4^+^ regulatory T cells, MDSC, TAM, B cells, and Th17 (Spearman’s correlation test, *p* <0.05) (Figure 12A), but not correlated with M2 macrophages. Besides, the *UCA1* expression levels were related to TAM, CAFs, CD8 cells, MDSC, and M2 macrophages (Spearman’s correlation test, *p* <0.05) (Figure 12B), but not related to CD4 cells, Macrophages, NK cells, NK cells, Th17, and Treg. These findings strongly suggest that *CCL5* could play a specific role in prognosis and immune activation in LAML, instead of *UCA1* could play a specific role in prognosis and immune inhibition in LAML.

### Verified the Expression of Hub Genes in Independent Clinical Samples

**FIGURE 13:**
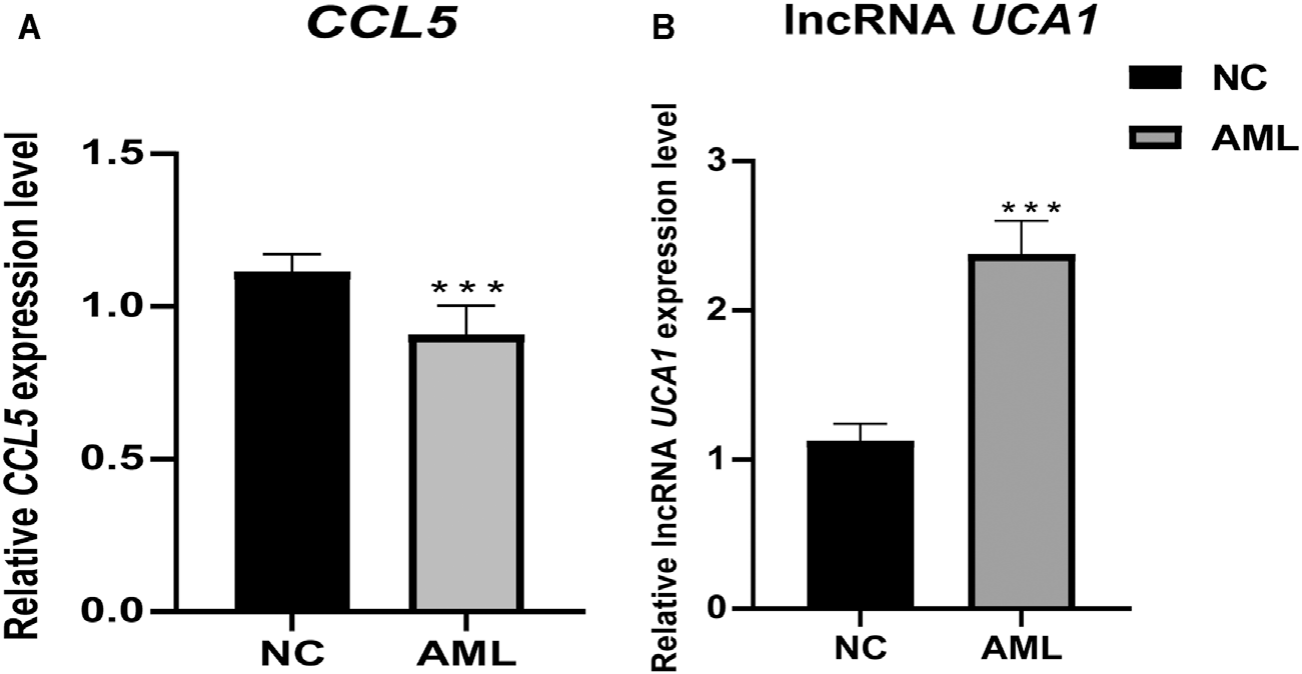
Differential expression of pivotal genes in clinical samples between AML and healthy individuals. Clinical validation results showed that the expression of CCL5 and lncRNA UCA1 was significantly lower and higher in primary AML, respectively. ***, *p* <0.001.

In our crucial gene expression validation results in 24 independent clinical samples (12 AML vs. 12 healthy individuals), we found that *CCL5* was significantly low expressed in the AML patient group. At the same time, lncRNA *UCA1* was significantly highly expressed in the AML patient group, which is consistent with our transcriptome sequencing results, demonstrating that these two key genes may serve as potential biological markers of diagnostic, targeted therapeutic (Figure 13).

## Discussion

Acute myeloid leukemia is a malignant clonal disease of the myeloid hematopoietic system with a highly heterogeneous (Kantarjian H. et al., 2021). AML is represented as critical and progressing rapidly, and with high mortality and poor prognosis in clinical. Therefore, finding effective biomarkers is essential for the diagnosis, treatment, and clinical outcome prediction of AML. In the past few decades, the research on AML has been mainly based on molecular characteristics, morphological characteristics, and immunological characteristics to explore tumor-associated protein-coding genes (Kantarjian H. M. et al., 2021). However, based on the development of high-throughput sequencing technology, researchers have identified and classified various non-coding RNAs that affect the pathogenesis of AML (Estey, 2020). In 2011, Salmena et al. proposed the scientific hypothesis of competitive endogenous RNA (ceRNA). As lncRNA, mRNA, cirRNA, and pseudogenes could be competitively combined with miRNA through miRNA response elements, affecting miRNA-mediated gene silencing (Salmena et al., 2011). Therefore, research on the pathogenic mechanism of ceRNA has been widely carried out in many malignant tumors such as Lung Adenocarcinoma, Liver Cancer, Colorectal Cancer, and et al. (Wang et al., 2020a; Di Palo et al., 2020; Sun et al., 2020) An increasing number of studies have recently demonstrated that the regulatory mechanisms of some ceRNAs in AML are receiving increasing attention. For example, S*BF2-AS1, CCAT1, UCA1* regulate *miR-188-5p, miR-155, miR-125a*, thereby up-regulating the expression of target genes *ZFP9, c-Myc,* and *HK2* promotes the proliferation of AML cells (Chen et al., 2016; Zhang et al., 2018; Tian et al., 2019). However, there is still limited research on systematically regulating lncRNA-miRNA-mRNA in AML and even less investigation of the ceRNA network based on transcriptome sequencing. This study mainly used transcriptome sequencing data of the AML and normal samples to obtain the differentially expressed lncRNA, miRNA, and mRNA expression data. Then we constructed the potential interactions network based on the regulatory relationship between the miRNA target gene’s prediction results and the differentially expressed lncRNAs and mRNAs. Finally, we found that hub genes as *CCL5* and *UCA1* could impact the prognosis of AML.

Long non-coding RNAs are RNA transcripts with over 200 nucleotides in length and lack protein-coding ability, which can be aberrantly expressed in various cancers (Zhang et al., 2020). Currently, increasing evidence suggests that dysregulation of lncRNAs may contribute to the development and progression of leukemia—research targeting lncRNAs showed clinical potential as a biomarker and therapeutic effect in hematological malignancies (Bhat et al., 2020). In particular, lncRNAs can interact with miRNAs as a competing endogenous RNA to regulate gene expression at epigenetic, transcriptional, and post-transcriptional levels. Undoubtedly, it would be of great importance to investigate lncRNAs as biomarkers and therapeutic targets for new individualized treatments in AML. In the current study, we obtained 12 DElnRNAs by combining transcriptome sequencing data and predictive analysis of miRNAs target genes, and the lncRNA *UCA1* was identified with a significant prognostic value in AML. Urothelial carcinoma-associated 1 (*UCA1*) is a long-stranded non-coding RNA that was initially identified in bladder metastatic cell carcinoma (Wang et al., 2006). Several studies have shown that lncRNA *UCA1* plays an essential role in the growth, differentiation, and metastasis of various tumor cells and is associated with chemoresistance in many tumors (Chen et al., 2021). However, to our knowledge, the biological function of lncRNA *UCA1* in AML is still limited. Research reported that C/EBPα (CCAAT/enhancer-binding protein) and C/EBPα-p30 could negatively regulate lncRNA *UCA1* expression by binding to the promoter and highly expressed AML cases with cytogenetically normal and carrying biallelic CEBPA mutation. Also, lncRNA *UCA1* could be an oncogene by inhibiting the expression of p27kip1 from maintaining the proliferation of AML cells, suggesting that lncRNA *UCA1* could be a novel diagnostic biomarker and a potential therapeutic target for AML in AML with CEBPA mutation (Hughes et al., 2015). Besides, another study has discovered that the expression of lncRNA *UCA1* was up-regulated after adriamycin chemotherapy, suggesting that lncRNA *UCA1* could act as an anti-chemotherapy agent. Conversely, knockdown of lncRNA *UCA1* could inhibit glycolysis through the miR-125a/HK2 pathway and reduce chemotherapy tolerance in childhood AML (Junge et al., 2017). In addition, lncRNA *UCA1* was found to be up-regulated in human myeloid leukemia cell lines (K562 and HL60), while knockdown of lncRNA *UCA1* inhibited the survival, migration, and invasion of myeloid leukemia cells *in vitro* and contributed to their apoptosis. The results of target gene validation studies showed that lncRNA *UCA1* could bind to miR-126 and downregulate miR-126 expression, thus forming a lncRNA *UCA1*/miR-126/RAC1 CeRNA regulatory network (Sun et al., 2018). In a clinical validation study of lncRNA *UCA1* expression in pediatric AML, elevated lncRNA *UCA1* and suppressed miR-204 expression were significantly correlated. Further *in vitro* AML cell line studies confirmed that silencing of lncRNA *UCA1* inhibited the proliferative capacity of AML cells. In contrast, overexpression of lncRNA UCA1 reversed its inhibitory effect on AML cells. The target validation study results suggested that lncRNA *UCA1* may play an oncogenic role in AML by indirectly enhancing SIRT1 expression by suppressing miR-204 *via* sponge interaction (Liang et al., 2020). Notably, it has been found that lncRNA *UCA1* can promote AML cell proliferation by inducing autophagy, and lncRNA *UCA1* could act as a sponge for binding miR-96–5p, thereby indirect inhibiting the targeting of ATG7, which could help us to understand further the molecular mechanism mediated by lncRNA *UCA1* responsible for the induction of autophagy in AML (Li J. et al., 2020). However, in recent years, it has also been found that knockdown of lncRNA *UCA1* induced apoptosis and inhibited the proliferation in AML cells (U937 and HL60), indicating lncRNA *UCA1* could meditate the AML process by regulating the miR-296–3p/Myc axis (Li J. J. et al., 2020).

Chemokines and their receptors are critical in the pathogenesis of AML, and they can regulate tumorigenesis by attracting anti-tumor and pro-tumor leukocytes to the tumor microenvironment, many of which are associated with regulation of the tumor microenvironment (Chow and Luster, 2014). *CCL5* is an important cytokine with physiological regulation of immune cell migration, also identified as a unique chemokine-releasing cluster in AML (Suenaga et al., 2016). Some studies have found that *CCL5* contributes to progression, metastasis, and even chemotherapy resistance in several tumors (Pasquier et al., 2018; Xiang et al., 2019). The cytokine *CCL5* could protect AML cells from TKI-mediated cell death and induce treatment resistance (Waldeck et al., 2020). Importantly, our study also found that *CCL5* expression showed a significant positive correlation with various immune signatures. Clinical observation has found that current conventional chemotherapy regimens (adriamycin combined with cytarabine) do not entirely inhibit *CCL5* expression, proposing that inhibition of chemokine *CCL5* expression could substantially improve the prognosis of AML (Yazdani et al., 2020). An analysis of a comprehensive study based on the GEO and TCGA-LAML databases found that *CCL5* is significantly highly expressed in AML patients and is a risk factor for poor prognosis (Chen et al., 2019). Recently, a study has demonstrated that *CCL5* is considerably overexpressed in FLT3-TKIs resistant AML cell lines, and the molecular mechanism might be related to the regulatory affection of stress-inducible protein p38 MAPK JNK (Pulliam et al., 2016). In the tumor microenvironment, tumor cells recruit normal cells by secreting *CCL5* and training them to become immunosuppressive tumors (Casagrande et al., 2019). Besides, *CCL5* supports pro-angiogenic activity by increasing endothelial cell migration, proliferation, neointima formation, and Vascular Endothelial Growth Factor (VEGF) secretion (Wang et al., 2015). Similarly, *CCL5* binds its receptor CCR5 with high affinity and promotes tumor progression through the CCL5/CCR5 signaling axis (Aldinucci et al., 2020). It has been proposed that CCL5/CCR5 axis may promote tumor development in multiple ways, such as growth factors, stimulating angiogenesis, regulating the extracellular matrix, inducing recruitment of additional stromal cells and inflammatory cells, and participating in immune evasion mechanisms (Aldinucci and Colombatti, 2014). The AML microenvironment includes complex interactions between immunosuppressive cell types, cytokines, and surface stimulatory molecules (Lamble et al., 2020). A study reported that AML cells expressing *CCL5* and CCR5 further profoundly affect AML progression via the CCL5/CCR5 axis (Binder et al., 2018). *CCL5* released from human AML cells has been shown to affect immune cell migration and has been shown to play an essential role in the chemotaxis and homing of AML cells (Weitzenfeld and Ben-Baruch, 2014; Park et al., 2015). A study recently focused on single-cell profiles of multiple immune phenotypes in the acute myeloid leukemia microenvironment. The research results showed that AML patients with specific MACRO subgroup gene profiles and *CCL5* expression were found to be significantly associated with poor overall survival (Guo et al., 2021). Furthermore, a study comparing the different stages of AML found that *CCL5* had higher levels in disease progression and was associated with GVHD development than the first diagnosis. Patients with low levels of *CCL5* were more likely to be associated with a subtype with a good prognosis and response to Immunotherapy (Merle et al., 2020). In addition, AML is an aggressive disease, the other research report that *CCL5* has been found to increase the proliferation of leukemic cells by binding to CCR3 as an inflammatory compartmentalizer (Bruserud et al., 2007). Besides, *CCL5* is physiologically a regulator of immune cell migration and is currently identified as a unique chemokine in AML and a critical mediator in inducing resistance to FLT-ITD tyrosine kinase inhibitors (Waldeck et al., 2020).

Studies have reported that *HGF* is a powerful and potent angiogenic factor, initially identified as a stimulator of hepatocyte growth *in vitro* and later characterized as a cytokine with mitogenic, motile, and morphogenic effects, involved in various normal developmental and homeostatic processes of the body (Jiang et al., 1999). Subsequently, *HGF* was found to be involved in cancer invasion and metastasis in a variety of solid tumors and was associated with poor prognosis (Kentsis et al., 2012; Spina et al., 2015). S. Verstovsek et al. found that plasma *HGF* levels were significantly higher in AML patients compared to healthy, and the results of multivariate analysis showed that elevated plasma *HGF* levels as a prognostic factor in AML were associated with shorter survival in AML patients, but they suggested that this variation in results may be due to differences in the samples (plasma rather than serum) (Verstovsek et al., 2001). In addition, *HGF* can induce VEGF production and thus play an important role in the pathophysiology of leukemia (Smolej et al., 2007; Reikvam et al., 2012). Furthermore, *HGF* as an angiogenic agent may be one of the key factors contributing to bleeding, which is a fatal event in AML patients (Anderlini et al., 1996; Verstovsek et al., 2001). Recently, several studies have demonstrated that *HGF* can promote the proliferation and migration of leukemic cells through the activation of *MET* receptors *via* autocrine and paracrine pathways (Kentsis et al., 2012; Guo et al., 2016). Also, it was found that *HGF* is the second most significantly overexpressed gene in the comparison between APL and non-APL (Gutiérrez et al., 2005). However, based on our findings, we found that *HGF* was a protective factor for survival in AML patients when analyzed univariately, but not significantly when analyzed multivariate. This result coincides with previous studies reporting that *HGF* may indirectly affect the prognosis of AML patients, whereas the prognostic effect of *HGF* was no longer significant after adjusting for the effects of certain factors in multivariate analysis, which likewise provides a possibility for related studies to further elucidate the combination of cytokines associated with *HGF* and thus reveal the development of AML (Fuhrmann-Benzakein et al., 2000).

Platelet-responsive proteins (THBS) are a family of multifunctional extracellular matrix proteins involved in inter and intracellular interactions and are associated with Platelet Aggregation, Angiogenesis, and Tumorigenesis (Huang et al., 2017). *THBS1* was the first member of the THBS to be identified and is thought to be an endogenous inhibitor of angiogenesis (Jeanne et al., 2015). Previous studies have shown that *THBS1* is highly expressed in various tumors (Jayachandran et al., 2014; Pal et al., 2016; Kamijo et al., 2020; Zhang et al., 2021). Furthermore, Lidan et al. have found that *THBS1* was lowly expressed in AML patients, which may be induced by promoter methylation, and patients with low *THBS1* expression had a shorter survival time; furthermore, Allogeneic Hematopoietic Stem Cell transplantation can overcome the adverse effects mediated by low *THBS1* expression (Zhu et al., 2019). Indeed, the above implies a potential function of *THBS1* in tumorigenesis. However, the role of *THBS1* expression in AML has still been less reported. In our study, we found the same confirmation of low *THBS1* expression in the bone marrow of AML patients, and this result was validated by previous studies where *THBS1* expression was significantly lower, but methylation was higher in AML patients, and methylation of the *THBS1* gene was associated with AML and prognosis, while hypomethylating agent treatment resulted in upregulation of *THBS1* expression and lower methylation levels (Zhu et al., 2019). This result suggested that elevated *THBS1* by hypomethylating agent treatment may be an important therapeutic direction for future AML treatment.

The Tumor Microenvironment (TME) includes multiple immune and stromal cell types, Secreted Extracellular Components, and An Intra-Tumor Environment representing Chronic Inflammation, Immunosuppression, and Pro-Angiogenesis (Pitt et al., 2016; Wu and Dai, 2017). AML blasts can adapt and grow in the myeloid environment and therefore have a high potential to evade host immune surveillance, allowing leukemic cells to be more susceptible to immune escape and more likely to develop resistance to external stimuli such as chemotherapy (Wang et al., 2020b). In recent years, despite many research results in the field of immune cell infiltration in solid tumors and the development of immune checkpoint inhibitors such as anti-PD-1, CTLA-4 for the Treatment of Renal Cell Carcinoma, Metastatic Melanoma, and Non-Small Cell Lung Cancer(Tawbi et al., 2018). However, immune signature and TME-related studies related to the pathogenesis and prognosis of AML are still very limited. Therefore, there has been considerable interest in characterizing leukemia-associated TME markers. Our analysis also observed that the expression of lncRNA *UCA1* and *CCL5* was closely associated with immune infiltration. Interestingly, *CCL5* and lncRNA *UCA1* had opposite functions, with *CCL5* significantly and positively correlated with immune score and stromal score, whereas lncRNA *UCA1* was negatively correlated with the immune score. Other immune signatures revealed that *CCL5* expression significantly increased the scores of 10 immune cells (Macrophages, NK cells, CD8^+^ T cells, CAFs, Treg, CD4^+^ regulatory T cells, MDSC, TAM, B cells, and Th17), while lncRNA *UCA1* significantly decreased the scores of TAM, MDSC, and M2 macrophages. *CCL5* is secreted by T cells, Platelets, Macrophages, Endothelial cells, and Fibroblasts and stimulates the migration of T cells and monocytes to drive apoptosis of tumor-infiltrating T cells; therefore, it was found that reducing the expression of *CCL5* may have a beneficial effect on various tumors (Bai et al., 2020; Korbecki et al., 2020; Nakamura et al., 2021). Besides, there are very limited studies on the involvement of lncRNA *UCA1* in tumor immunity, among which studies found that lncRNA *UCA1* as an oncogene could suppress the host immune system by up-regulating the PDL1 level in gastric cancer cells (Wang J.-D. et al., 2019). And targeting lncRNA UCA1 induced intratumoral interferon gamma-dependent programmed cell death ligand 1 expression *in vivo*, thereby synergistically enhancing the anti-bladder cancer activity of immune checkpoint blockade of PD-1 (Zhen et al., 2018). However, as we know, this is the first time report that the expression of lncRNA *UCA1* is associated with immunosuppression in AML, and these new findings will provide new insights to reveal immune infiltration-related CeRNA regulatory networks.

Nevertheless, this study also has some limitations. Firstly, we should notice that AML is a complex and heterogeneous disease, so different subtypes, individuals, and genetic characteristics may lead to various disease processes. Therefore, this study’s results need to be clarified based on a larger sample and more accurate diagnostic typing. In addition, only part of lncRNAs may act as classic miRNA “sponges,” while the biological functions of more lncRNAs are still unclear, their targeting relationship with miRNAs and the functions in post-transcriptional regulation still need to be further verified. We will further investigate the molecular mechanisms of the identified hub genes in future studies. Meanwhile, we will further investigate the protein levels in cell lines and *in vivo* models.

In summary, we identified *CCL5* and lncRNA *UCA1* as key regulators in the AML transcriptome that significantly affect prognosis by constructing the potential interactions network. Besides, we performed a more far-reaching predictive study of the impact of key genes using multiple bioinformatics analysis tools and further investigated the association between *CCL5* and lncRNA *UCA1* and AML-related immune infiltration. Furthermore, we found that the *CCL5* was lower expressed in the clinical samples of AML, while lncRNA *UCA1* was highly expressed in the clinical samples of AML. Based on this study’s findings, we propose that *CCL5* and lncRNA *UCA1* could be possible biomarkers for predicting survival prognosis in AML. However, further studies are needed to validate these findings.

## Data Availability

Publicly available datasets were analyzed in this study. This data can be found in TCGA-LAML (https://portal.gdc.cancer.gov/) and GEO datase (https://www.ncbi.nlm.nih.gov/geo/query/acc.cgi?accGSE114868).

## Supplementary Material

Supplementary Figure 1 The flow chart of study design.

Supplementary Figure 2 The PCA of the samples between different groups. PCA results show that all the samples from AML and normal are significantly distinguished into two groups. PCA, principal components analysis.

Supplementary Figure 3 The potential interactions network constructed by the interacted lncRNAs, DEmiRNAs, and mRNAs. The turquoise dots represent the interacted mRNAs in the potential interactions network with a score of >0.5; the purple rhombus represents the interacted lncRNAs in the potential interactions network with a score of >0.5; the yellow triangle represents the DEmiRNAs in the potential interactions network.

Supplementary Table 1 Clinical information of the collected samples in transcriptome sequencing.

Supplementary Table 2 Differentially expressed mRNAs between AML and normal samples.

Supplementary Table 3 Differentially expressed miRNAs between AML and normal samples.

Supplementary Table 4 Differentially expressed lncRNAs between AML and normal samples.

Supplementary Table 5 Top 10 GO analysis results in the potential interactions network.

Supplementary Table 6 Top 20 KEGG enrichment results in the ceRNA network.
