# Supplementary material for: Identification of Potential Novel Prognosis-Related Genes Through Transcriptome Sequencing, Bioinformatics Analysis, and Clinical Validation in Acute Myeloid Leukemia
Source: Front Genet. 2021 Oct 29;12:723001. doi: 10.3389/fgene.2021.723001 (PMC8585857; doi:10.3389/fgene.2021.723001)
Supplement: Supplementary file 3 [file Table1.DOCX]

**Supplementary Table 1** **Clinical information of the collected samples in transcriptome sequencing**

| **Samples** | **Age** | **Gender** | **Leukocyte** | **Hemoglobin** | **Platelet** | **Diagnosis**  **(FAB classification systems)** | **Cytogenetics risk** |
| --- | --- | --- | --- | --- | --- | --- | --- |
| AML 1 | 50 | Male | 38.27 | 71 | 98 | AML-M5 | Poor |
| AML 2 | 75 | Male | 1.8 | 79 | 7 | AML-M4b | Favorable |
| AML 3 | 65 | Male | 35.6 | 95 | 15 | AML-M2a | Intermediate/normal |
| AML 4 | 51 | Male | 8.3 | 104 | 47 | AML-M5b | Poor |
| AML 5 | 55 | Female | 139 | 71 | 17 | AML-M1 | Poor |
| AML 6 | 27 | Male | 107.66 | 95 | 50 | AML-M1 | Poor |
| AML 7 | 48 | Male | 78.3 | 1.41 | 30.07 | AML-M1 | Intermediate/normal |
| AML 8 | 60 | Female | 20.76 | 105 | 41 | AML-M2 | Favorable |
| AML 9 | 34 | Female | 17 | 74 | 10 | AML-M2 | Intermediate/normal |
| AML 10 | 18 | Male | 221.7 | 99 | 65 | AML-M5 | Poor |
| Normal 1 | 37 | Female | - | - | - | - | - |
| Normal 2 | 39 | Female | - | - | - | - | - |
| Normal 3 | 35 | Male | - | - | - | - | - |
| Normal 4 | 30 | Male | - | - | - | - | - |
| Normal 5 | 33 | Male |  |  |  | - | - |
