# Supplementary figures and images for: Identification of Potential Novel Prognosis-Related Genes Through Transcriptome Sequencing, Bioinformatics Analysis, and Clinical Validation in Acute Myeloid Leukemia
Source: Front Genet. 2021 Oct 29;12:723001. doi: 10.3389/fgene.2021.723001 (PMC8585857; doi:10.3389/fgene.2021.723001)

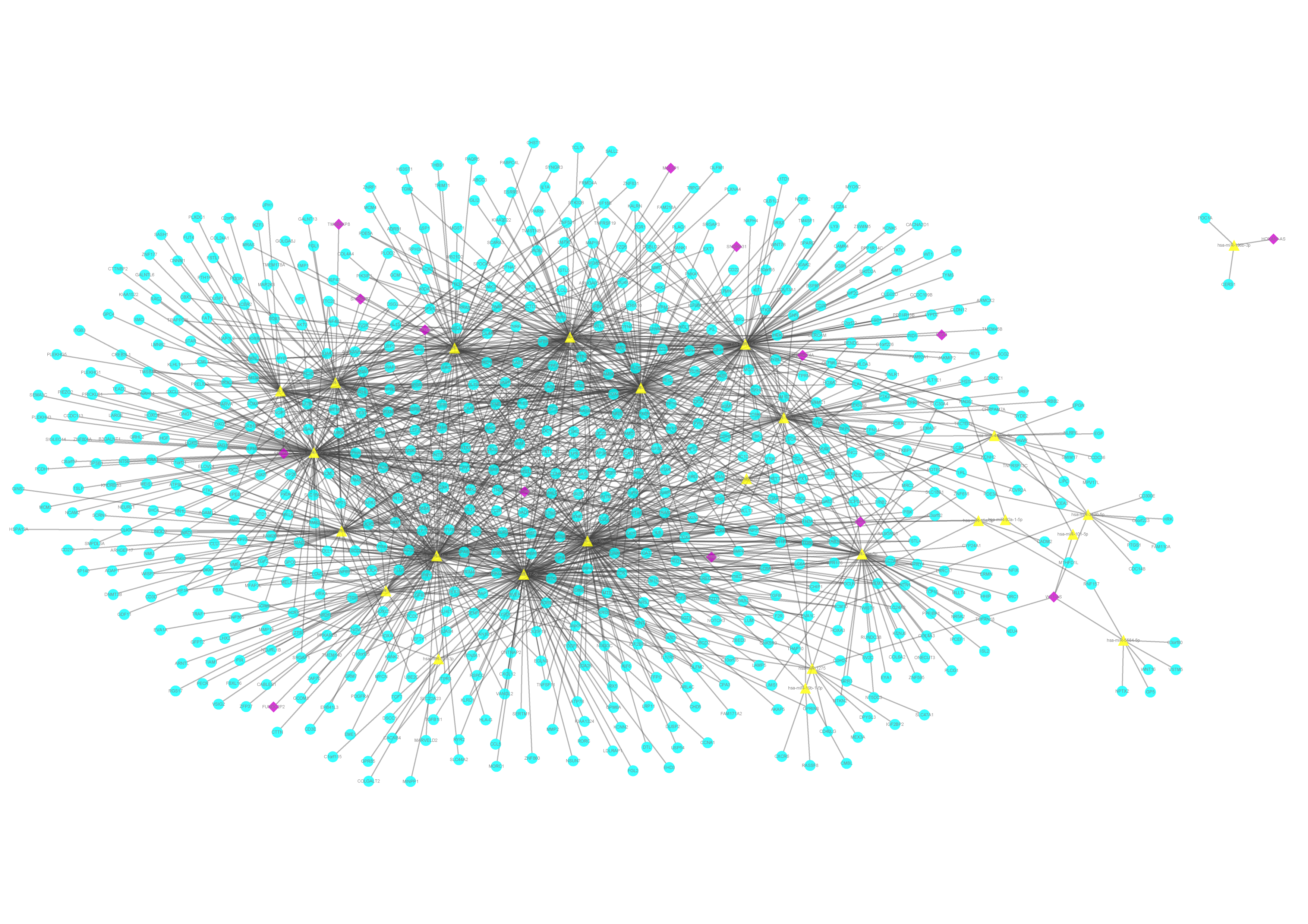

Supplement: Supplementary file 4 [file Image3.TIF]

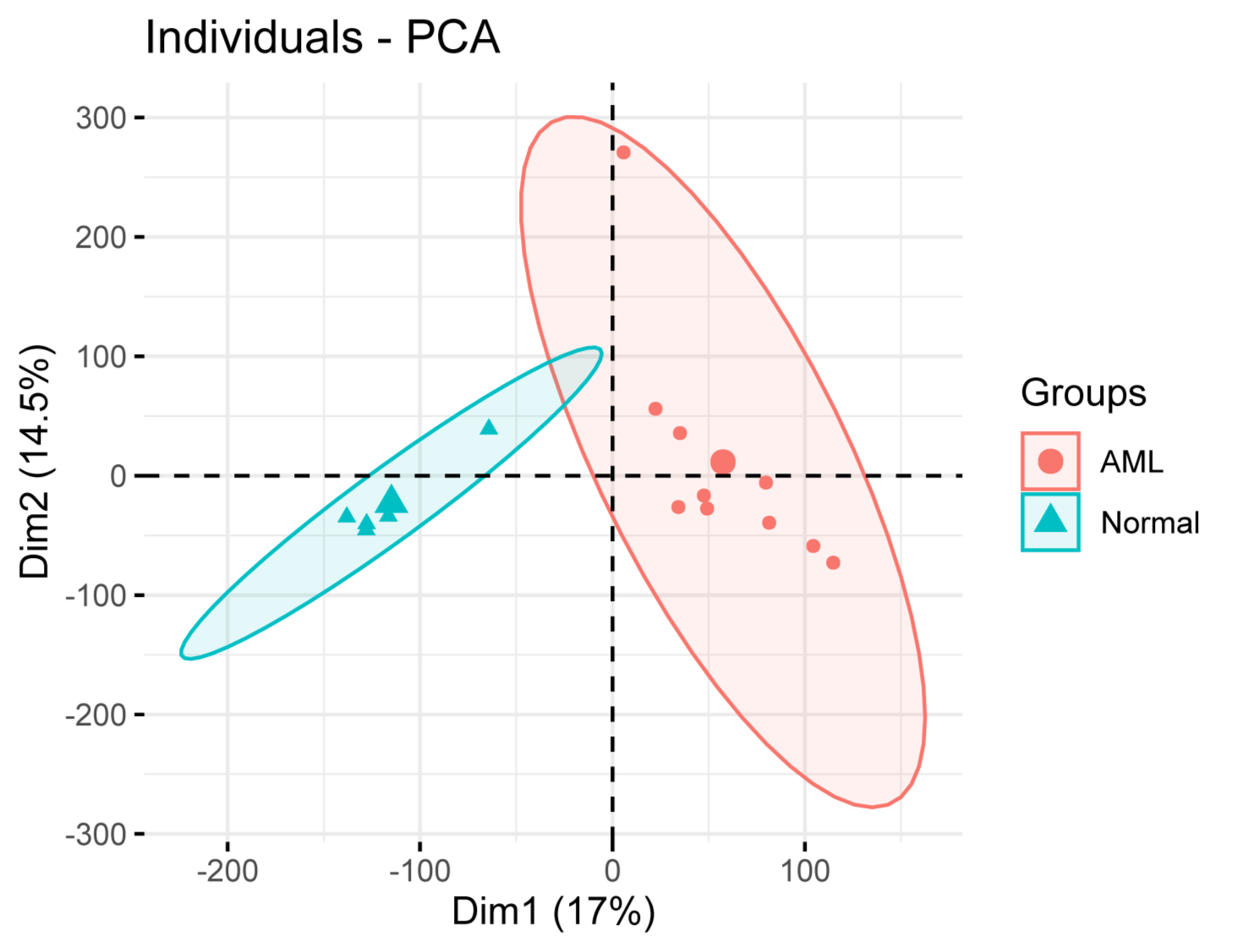

Supplement: Supplementary file 5 [file Image2.TIF]

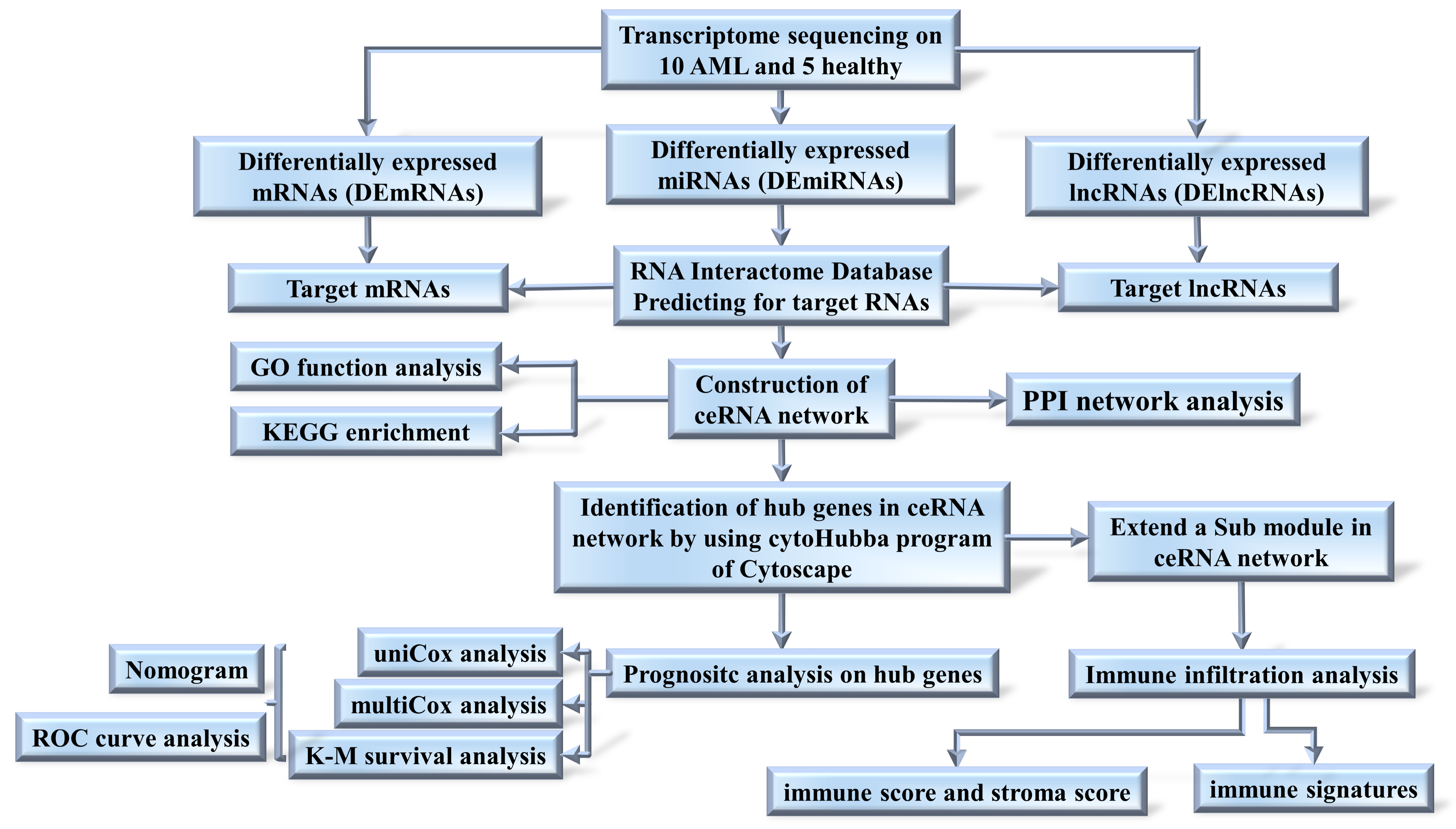

Supplement: Supplementary file 6 [file Image1.TIF]
