# Supplementary material for: Identification of Potential Novel Prognosis-Related Genes Through Transcriptome Sequencing, Bioinformatics Analysis, and Clinical Validation in Acute Myeloid Leukemia
Source: Front Genet. 2021 Oct 29;12:723001. doi: 10.3389/fgene.2021.723001 (PMC8585857; doi:10.3389/fgene.2021.723001)
Supplement: Supplementary file 7 [file Table5.DOCX]

**Supplementary Table 5 Top 10 GO analysis results in the interactions network**

| **Category** | **Term** | **Count** | ***P*_Value** | **adj_pval** |
| --- | --- | --- | --- | --- |
| BP | positive regulation of transcription from RNA polymerase II promoter | 70 | 8.80E-08 | 1.59E-04 |
| BP | positive regulation of gene expression | 30 | 9.56E-08 | 1.73E-04 |
| BP | positive regulation of cell migration | 23 | 9.21E-07 | 0.001664 |
| BP | angiogenesis | 25 | 2.01E-06 | 0.003639 |
| BP | positive regulation of phosphatidylinositol 3-kinase signaling | 13 | 3.22E-06 | 0.005825 |
| BP | transcription from RNA polymerase II promoter | 41 | 4.69E-06 | 0.008483 |
| BP | steroid hormone mediated signaling pathway | 12 | 5.27E-06 | 0.009533 |
| BP | anterior/posterior pattern specification | 14 | 5.58E-06 | 0.010087 |
| BP | wound healing | 14 | 5.58E-06 | 0.010087 |
| BP | intracellular receptor signaling pathway | 10 | 6.50E-06 | 0.011745 |
| CC | cell-cell junction | 26 | 2.27E-09 | 3.17E-06 |
| CC | plasma membrane | 205 | 8.65E-08 | 1.21E-04 |
| CC | receptor complex | 17 | 1.16E-05 | 0.016167 |
| CC | lamellipodium | 18 | 5.83E-05 | 0.081478 |
| CC | focal adhesion | 30 | 1.67E-04 | 0.232873 |
| CC | integral component of plasma membrane | 77 | 2.00E-04 | 0.278903 |
| CC | extracellular space | 74 | 2.02E-04 | 0.282299 |
| CC | proteinaceous extracellular matrix | 23 | 2.54E-04 | 0.354575 |
| CC | external side of plasma membrane | 19 | 6.41E-04 | 0.892011 |
| CC | platelet alpha granule lumen | 9 | 6.88E-04 | 0.956661 |
| MF | sequence-specific DNA binding | 54 | 8.78E-12 | 1.34E-08 |
| MF | transcription factor activity, sequence-specific DNA binding | 66 | 8.54E-07 | 0.001303 |
| MF | RNA polymerase II core promoter proximal region sequence-specific binding | 26 | 1.64E-06 | 0.002509 |
| MF | steroid hormone receptor activity | 11 | 2.88E-05 | 0.04389 |
| MF | ligand-activated sequence-specific DNA binding | 9 | 3.51E-05 | 0.053527 |
| MF | protein binding | 362 | 2.57E-04 | 0.391577 |
| MF | Ras guanyl-nucleotide exchange factor activity | 14 | 2.71E-04 | 0.412379 |
| MF | growth factor activity | 16 | 8.26E-04 | 1.252063 |
| MF | phosphatidylinositol-4,5-bisphosphate 3-kinase activity | 9 | 0.001716 | 2.585581 |
| MF | extracellular matrix binding | 6 | 0.002152 | 3.233375 |

**Note:** BP: biological processes; CC, cellular components; MF, molecular functions.
