# Supplementary material for: Identification of Potential Novel Prognosis-Related Genes Through Transcriptome Sequencing, Bioinformatics Analysis, and Clinical Validation in Acute Myeloid Leukemia
Source: Front Genet. 2021 Oct 29;12:723001. doi: 10.3389/fgene.2021.723001 (PMC8585857; doi:10.3389/fgene.2021.723001)
Supplement: Supplementary file 9 [file Table6.DOCX]

**Supplementary Table 6 Top 20 KEGG enrichment results in the interactions network**

| **Term** | **Count** | ***P*_Value** | **FDR** |
| --- | --- | --- | --- |
| hsa05200: Pathways in cancer | 44 | 2.39E-09 | 3.08E-06 |
| hsa05205: Proteoglycans in cancer | 28 | 3.97E-08 | 5.12E-05 |
| hsa04015: Rap1 signaling pathway | 25 | 4.95E-06 | 0.006383 |
| hsa04510: Focal adhesion | 24 | 1.16E-05 | 0.014934 |
| hsa05217: Basal cell carcinoma | 11 | 5.85E-05 | 0.075446 |
| hsa05166: HTLV-I infection | 25 | 1.17E-04 | 0.150125 |
| hsa04810: Regulation of actin cytoskeleton | 22 | 1.42E-04 | 0.182923 |
| hsa04014: Ras signaling pathway | 23 | 1.46E-04 | 0.187523 |
| hsa05202: Transcriptional misregulation in cancer | 19 | 1.67E-04 | 0.214789 |
| hsa04360: Axon guidance | 15 | 7.04E-04 | 0.903254 |
| hsa04512: ECM-receptor interaction | 12 | 8.36E-04 | 1.072225 |
| hsa04151: PI3K-Akt signaling pathway | 28 | 9.48E-04 | 1.21468 |
| hsa04024: cAMP signaling pathway | 19 | 0.00131 | 1.675588 |
| hsa04060: Cytokine-cytokine receptor interaction | 21 | 0.002425 | 3.08168 |
| hsa04916: Melanogenesis | 12 | 0.002631 | 3.338493 |
| hsa04974: Protein digestion and absorption | 11 | 0.003206 | 4.054441 |
| hsa04010: MAPK signaling pathway | 21 | 0.003832 | 4.828249 |
| hsa04310: Wnt signaling pathway | 14 | 0.004435 | 5.568924 |
| hsa05214: Glioma | 9 | 0.004989 | 6.243776 |
